# *In vitro* and *in vivo* metabolism of psilocybin’s active metabolite psilocin

**DOI:** 10.3389/fphar.2024.1391689

**Published:** 2024-04-29

**Authors:** Jan Thomann, Karolina E. Kolaczynska, Oliver V. Stoeckmann, Deborah Rudin, Patrick Vizeli, Marius C. Hoener, Christopher R. Pryce, Franz X. Vollenweider, Matthias E. Liechti, Urs Duthaler

**Affiliations:** ^1^ Division of Clinical Pharmacology and Toxicology, Department of Pharmaceutical Sciences, University of Basel, Basel, Switzerland; ^2^ Division of Clinical Pharmacology and Toxicology, Department of Biomedicine, University Hospital Basel, Basel, Switzerland; ^3^ Neuroscience Research, Pharma Research and Early Development, Roche Innovation Center Basel, F. Hoffmann-La Roche Ltd, Basel, Switzerland; ^4^ Department of Psychiatry, Psychotherapy and Psychosomatics, Preclinical Laboratory for Translational Research Into Affective Disorders, University of Zurich, Zurich, Switzerland; ^5^ Department of Psychiatry, Psychotherapy and Psychosomatics, Neurophenomenology and Consciousness, University of Zurich, Zurich, Switzerland; ^6^ Institute of Forensic Medicine, Department of Biomedical Engineering, University of Basel, Basel, Switzerland; ^7^ Institute of Forensic Medicine, Health Department Basel-Stadt, Basel, Switzerland

**Keywords:** psychedelics, psilocybin, metabolism, cytochrome P450 (CYP), pharmacokinetics, liver microsomes, recombinant enzymes, 5-HT receptor

## Abstract

*In vivo*, psilocybin is rapidly dephosphorylated to psilocin which induces psychedelic effects by interacting with the 5-HT_2A_ receptor. Psilocin primarily undergoes glucuronidation or conversion to 4-hydroxyindole-3-acetic acid (4-HIAA). Herein, we investigated psilocybin’s metabolic pathways *in vitro* and *in vivo*, conducting a thorough analysis of the enzymes involved. Metabolism studies were performed using human liver microsomes (HLM), cytochrome P450 (CYP) enzymes, monoamine oxidase (MAO), and UDP-glucuronosyltransferase (UGT). *In vivo*, metabolism was examined using male C57BL/6J mice and human plasma samples. Approximately 29% of psilocin was metabolized by HLM, while recombinant CYP2D6 and CYP3A4 enzymes metabolized nearly 100% and 40% of psilocin, respectively. Notably, 4-HIAA and 4-hydroxytryptophol (4-HTP) were detected with HLM but not with recombinant CYPs. MAO-A transformed psilocin into minimal amounts of 4-HIAA and 4-HTP. 4-HTP was only present *in vitro*. Neither 4-HIAA nor 4-HTP showed relevant interactions at assessed 5-HT receptors. In contrast to *in vivo* data, UGT1A10 did not extensively metabolize psilocin *in vitro*. Furthermore, two putative metabolites were observed. *N*-methyl-4-hydroxytryptamine (norpsilocin) was identified *in vitro* (CYP2D6) and in mice, while an oxidized metabolite was detected *in vitro* (CYP2D6) and in humans. However, the CYP2D6 genotype did not influence psilocin plasma concentrations in the investigated study population. In conclusion, MAO-A, CYP2D6, and CYP3A4 are involved in psilocin’s metabolism. The discovery of putative norpsilocin in mice and oxidized psilocin in humans further unravels psilocin’s metabolism. Despite limitations in replicating phase II metabolism *in vitro*, these findings hold significance for studying drug-drug interactions and advancing research on psilocybin as a therapeutic agent.

## 1 Introduction

Psychedelic mushrooms (e.g., *Psilocybe azurescens* or *Psilocybe mexicana*) and their associated mind- and consciousness-altering effects have been explored for over 3,000 years (Van Court et al., 2022). Most recently, psilocybin has been investigated for its therapeutic properties in several affective disorders including anxiety, treatment-resistant major depression, and cluster headache (Moreno et al., 2006; Sewell et al., 2006; Grob et al., 2011; Griffiths et al., 2016; Ross et al., 2016; Carhart-Harris et al., 2017; Johnson et al., 2017; Bogenschutz et al., 2018; Becker et al., 2022).

As a psychoactive alkaloid, psilocybin undergoes first-pass metabolism and acts as a prodrug. Upon oral ingestion, its terminal phosphate group (PO_4_) is rapidly cleaved by alkaline phosphates and non-specific esterases. This transforms psilocybin into psilocin (4-hydroxy-*N,N*-dimethyltryptamine), a more lipophilic molecule that can cross the blood-brain barrier more readily (Horita and Weber, 1961a; Horita and Weber, 1962; Eivindvik et al., 1989; Hasler et al., 1997) to produce its psychedelic effects via serotonin 5-HT_2A_ receptors (Vollenweider et al., 1998; Rickli et al., 2016). Psilocin is therefore the active agent that produces psilocybin’s mind-altering effects. Psilocin concentration peaks in plasma around 2 h after oral administration and the subjective effects last for approximately 6 h (Griffiths et al., 2016; Johnson et al., 2017; Holze et al., 2022a). In humans, psilocin plasma concentrations of approximately 15–20 ng/mL and an elimination half-life (t_1/2_) of 2–3 h were observed after a single oral dose of 25 mg psilocybin (Kolaczynska et al., 2021; Holze et al., 2022a). Psilocin is primarily excreted in urine, although only a minor fraction of administered psilocybin (1.5%) is eliminated as unconjugated psilocin in the first 24 h (Holze et al., 2022a). Psilocin is extensively glucuronidated to psilocin-O-glucuronide, a major urine metabolite. Approximately 20% of orally administered psilocybin is excreted as glucuronidated psilocin in humans within 24 h (Holze et al., 2022a). This conjugation is mainly catalyzed by UDP-glucuronosyltransferase (UGT) 1A10 and UGT1A9 which are highly expressed in the small intestine and liver, respectively (Sticht and Käferstein, 2000; Grieshaber et al., 2001; Hasler et al., 2002; Manevski et al., 2010; Kolaczynska et al., 2021). Concurrently, psilocin can undergo demethylation and oxidative deamination to form the intermediate metabolite 4-hydroxyindole-3-acetaldehyde (4-HIA), presumably catalyzed by monoamine oxidase (MAO). 4-HIA undergoes either reduction to 4-hydroxytryptophol (4-HTP) or oxidation to 4-hydroxindole-3-acetic acid (4-HIAA) (Kalberer et al., 1962; Hasler et al., 1997; Lindenblatt et al., 1998; Kolaczynska et al., 2021). 4-HIAA is, along with psilocin-O-glucuronide, another major urine metabolite. Around 33% of a psilocybin dose is renally excreted as 4-HIAA (Holze et al., 2022a). It is suggested that similar to the metabolic pathway of serotonin (5-hydroxytryptamine, 5-HT), aldehyde (ALDH) and alcohol dehydrogenase (ADH) might play a key role in transforming 4-HIA to 4-HIAA and 4-HTP, respectively (Svensson et al., 1999; Dinis-Oliveira, 2017). A further minor metabolic route is proposed to be the hydroxylation and oxidation of the indole moiety of psilocin to form an iminoquinone or *o*-quinone structure. It is hypothesized that this reaction is exerted by oxidative enzymes such as ceruloplasmin (copper carrying oxidase in human blood), cytochrome oxidases, or non-enzymatically by ferric oxide (Fe^3+^) but not MAO (Blaschko and Levine, 1960; Horita and Weber, 1961b; Dinis-Oliveira, 2017). However, the exact structure of the oxidized metabolite has not been experimentally elucidated. Moreover, to our best knowledge, the role of cytochrome P450 (CYP) enzymes in psilocybin’s metabolism has thus far not been demonstrated. The CYP superfamily is responsible for the oxidative metabolism of several psychoactive substances and has previously been shown to be involved in the metabolism of *N,N*-dimethyltryptamine (DMT), lysergic acid diethylamide (LSD), or 3,4-methylenedioxymethamphetamine (MDMA) (Vizeli et al., 2017; Luethi et al., 2019; Eckernäs et al., 2023).

Furthermore, the pharmacological activity or relevance of psilocybin’s metabolites needs to be assessed. Psilocin’s 5-HT receptor binding and receptor activation potency have been determined previously with relevant activity at human 5-HT_1A_, 5-HT_2A_, 5-HT_2B_, and 5-HT_2C_ receptors (Rickli et al., 2016). In addition, psilocin moderately inhibits the serotonin transporter (SERT) but not dopamine (DAT) or norepinephrine transporters (NET) (Rickli et al., 2016). However, it is not clear if any of psilocybin’s remaining metabolites display relevant pharmacological activity at 5-HT receptors or the monoamine transporters.

As psilocybin transforms from a recreational substance to a potential therapeutic agent, a comprehensive understanding of its pharmacological characteristics and metabolic breakdown is necessary. In this study, we aimed to characterize the metabolic pathways of psilocin *in vitro*, focusing on the involvement of CYP enzymes. Furthermore, we compared the *in vitro* findings to *in vivo* findings in both humans and mice.

## 2 Material and methods

### 2.1 Chemicals and reagents

LC-MS grade water, methanol, acetonitrile, and isopropanol were all purchased from Merck (Darmstadt, Germany). Formic acid, dimethyl sulfoxide (DMSO), potassium phosphate dibasic (K_2_HPO_4_), potassium phosphate monobasic (KH_2_PO_4_), and bovine serum albumin (BSA) were acquired from Sigma-Aldrich (Buchs, Switzerland).

### 2.2 Substrates, metabolites, inhibitors, and internal standards

Psilocin was purchased from Lipomed (Arlesheim, Switzerland), while 4-HIAA and 4-HTP were synthesized by ReseaChem (Burgdorf, Switzerland). The purity of all aforementioned analytes was >98%.

CYP substrates including tizanidine hydrochloride (CYP1A2) (S)-efavirenz (CYP2B6), paclitaxel (CYP2C8), flurbiprofen (CYP2C9), omeprazole (CYP2C19), and metoprolol (CYP2D6) were obtained from Toronto Research Chemicals (TRC; Toronto, Canada). CYP3A4 substrate midazolam was acquired from Lipomed, while chlorzoxazone (CYP2E1) was purchased from Sigma-Aldrich. Metabolites including hydroxy-tizanidine (OH-tizanidine; CYP1A2), 8-hydroxy-efavirenz (OH-efavirenz; CYP2B6), 6-α-hydroxy-paclitaxel (OH-paclitaxel; CYP2C8), 4-hydroxy-flurbiprofen (OH-flurbiprofen; CYP2C9), 5-hydroxy-omeprazole (OH-omeprazole; CYP2C19), α-hydroxy-metoprolol (OH-metoprolol; CYP2D6), and 6-hydroxy-chlorzoxazone (OH-chlorzoxazone; CYP2E1) were all purchased from TRC, while α-hydroxy-midazolam (OH-midazolam; CYP3A4) was obtained from Lipomed.

CYP inhibitors ticlopidine hydrochloride (CYP2B6), sulfaphenazole (CYP2C9) (+)-*N*-3-benzylnirvanol (CYP2C19), quinidine sulfate (CYP2D6), 4-methylpyrazole hydrochloride (CYP2E1), and ketoconazole (CYP3A4) were all purchased from Sigma-Aldrich. Furafylline (CYP1A2) was purchased from TRC while montelukast dicyclohexylamine (CYP2C8) was obtained from the European Directorate for the Quality of Medicines and Healthcare (Strasbourg, France).

The MAO-A and MAO-B substrate kynuramine dihydrobromide, its metabolite 4-hydroxyquinoline (4-HQ), and inhibitors clorgyline hydrochloride (MAO-A) and R-deprenyl hydrochloride (MAO-B) were all purchased from Sigma-Aldrich. *N,N*-dimethyltryptamine (DMT) and its metabolite indole-3-acetic acid (IAA) monosodium salt were acquired from Lipomed and Cayman Chemical (Ann Arbor, USA), respectively.

Internal standards (ISTD) psilocin-d_10_ and IAA-d_2_ were acquired from Sigma-Aldrich, while L-tryptophan-d_5_, tizanidine-d_4_, efavirenz-d_5_, paclitaxel-d_5_, flurbiprofen-d_3_, omeprazole-d_3_, metoprolol-d_6_, chlorzoxazone-d_3_, and midazolam-d_6_ were obtained from TRC. DMT-d_6_ was purchased from ReseaChem.

### 2.3 Metabolizing enzyme systems

Corning UltraPool human liver microsomes (HLM) 150 (20 mg/mL), human intestinal microsomes (HIM) pool (10 mg/mL) as well as recombinant human CYP enzymes (with P450 oxidoreductase, OR) including CYP1A2+OR (0.5 nmol), CYP2B6+OR (0.5 nmol), CYP2C8+OR (1.0 nmol), CYP2C9*1+OR (1 nmol), CYP2C19+OR (0.5 nmol), CYP2D6*1+OR (0.5 nmol), CYP2E1+OR + cytochrome b_5_ (1 nmol), CYP3A4+OR + cytochrome b_5_ (0.5 nmol), MAO-A (5 mg/mL), MAO-B (5 mg/mL), and UGT1A10 (5 mg/mL) were purchased from Corning Life Sciences B.V (Amsterdam, Netherlands). All microsomes and recombinant enzyme solutions were aliquoted and stored at −80 °C until further use. Co-factors including NADPH regenerating system solution A (25 mM NADP+, 66 mM glucose-6-phosphate, and 66 mM MgCl_2_ in water), NADPH regenerating system solution B (40 U/mL glucose-6-phosphate dehydrogenase in 5 mM sodium citrate), UGT reaction mix solution A (25 mM uridine 5′-diphospho-glucuronic acid in water), and UGT reaction mix solution B (250 mM Tris-HCl, 40 mM MgCl_2_, and 0.125 mg/mL alamethicin in water) were also obtained from Corning Life Sciences B.V.

### 2.4 Stocks and calibration standards

For psilocin, 4-HIAA, 4-HTP, 4-HQ, DMT, and CYP metabolites, calibration working solutions were prepared by serially diluting substance working mixes in a mix of equal parts of water and acetonitrile (for psilocin, 4-HIAA, and 4-HTP) or DMSO (for 4-HQ, DMT, and CYP metabolites). Subsequently, each calibration working solution was diluted 1:100 in the matrix of interest, namely, 0.1 M potassium phosphate buffer (pH 7.4, K_2_HPO_4_ and KH_2_PO_4_ in water, 5:1 v/v) containing 1.5% BSA, pooled mouse plasma, or pooled human plasma to cover the desired concentration range. Specific calibration ranges for each analyte are depicted in Supplementary Table S1.

### 2.5 Enzymatic inhibition assays

#### 2.5.1 Cytochrome P450 inhibition

The CYP inhibition assay using HLM was previously described by Luethi et al. (Luethi et al., 2019) and was adapted herein. In brief, psilocin, 4-HIAA, 4-HTP, or CYP substrate stocks were mixed (1:100 dilution) with NADPH regeneration system solution A (1:20 dilution), NADPH regenerating system solution B (1:100 dilution), and with or without CYP inhibitors (1:100 dilution) in 0.1 M PBS (pH 7.4) supplemented with 1.5% BSA. The tubes were vortexed and placed on a Thermomixer (Eppendorf, Hamburg, Germany) at 300 rpm and 37°C. Next, 50 μL of the mixture from each tube was taken out as a baseline sample and transferred to a corresponding matrix tube containing 150 μL ice cold ISTD solution. Directly after, microsomes (1:100 dilution, end concentration 0.2 mg/mL) or recombinant CYP enzymes (1:40 dilution, end concentration 0.0125 nM or 0.025 nM depending on the enzyme) were added to each reaction tube to initiate the metabolic reaction. The total volume of the reaction mixture was 500 μL. The mixture was sampled at 0, 30, 60, 120, 180, 210, and 240 min for psilocin, 4-HIAA, and 4-HTP, and 0, 30, 60, 90, 120, 150, and 180 min for CYP substrates. Afterward, the matrix tubes were vortexed and centrifuged for 30 min at 3,220 × g (Centrifuge 5810 R, Eppendorf). Samples were either directly analyzed by liquid chromatography tandem mass spectrometry (LC-MS/MS) or briefly stored at −20°C until analysis. The ISTD solution consisted of 20 ng/mL psilocin-d_10_ and 100 ng/mL tryptophan-d_5_ in methanol. A separate ISTD solution was prepared in acetonitrile for CYP substrates containing 100 ng/mL of tizanidine-d_4_, efavirenz-d_5_, flurbiprofen-d_3_, omeprazole-d_3_, metoprolol-d_6_, chlorzoxazone-d_3_, midazolam-d_6_, and 250 ng/mL paclitaxel-d_5_.

#### 2.5.2 Monoamine oxidase inhibition

The MAO inhibition assay was based on the assay described in section 2.5.1 and performed identically if not otherwise stated. Psilocin, 4-HIAA, 4-HTP, or MAO substrate kynuramine were incubated with recombinant MAO-A enzymes (1:100 dilution, end concentration 0.05 mg/mL), recombinant MAO-B enzymes (1:100 dilution, end concentration 0.05 mg/mL), or HLM (1:100 dilution, end concentration 0.2 mg/mL), and with or without MAO isoform-selective inhibitors clorgyline (MAO-A and HLM) or R-deprenyl (MAO-B). Sampling time points for psilocin, 4-HIAA, 4-HTP, and control conditions were adjusted to 0, 60, 120, 180, 240, and 300 min for MAO-A and 0, 60, 120, 180, and 240 min for MAO-B assays. DMT was incubated with recombinant MAO-A or MAO-B and sampling timepoints were 0, 60, 120, 180, and 240 min. A methanolic solution containing 5 ng/mL DMT-d_6_ and 500 ng/mL IAA-d_2_ was used as ISTD.

#### 2.5.3 Glucuronidation

*In vitro*, glucuronidation of psilocin was assessed using HLM, HIM, and recombinant human UGT1A10 enzymes. The assay described in section 2.5.1 was adjusted to suit the glucuronidation system. Psilocin or control substrate OH-efavirenz was diluted 1:100 in 0.1 M PBS (pH 7.4) without BSA and UGT reaction mix solution A (1:20 dilution) and B (1:100 dilution) were added as co-factors. HLM were diluted as described in section 2.5.1, while HIM or UGT1A10 enzymes were diluted 1:40 (end concentration 0.5 and 0.125 mg/mL, respectively) in the reaction mix. Sampling time points were adjusted to 0, 15, 30, 60, 90, 120, 180, and 240 min.

### 2.6 Human 5-HT receptor interactions

#### 2.6.1 5-HT receptor binding

Radioligand receptor binding assays of psilocin, 4-HIAA, and 4-HTP were assessed at the 5-HT_1A_, 5-HT_2A_, and 5-HT_2C_ receptors as previously described in detail by Luethi et al. (Luethi et al., 2018). In general, human embryonic kidney (HEK) 293 cell line membrane preparations overexpressing either the human 5-HT_1A_, 5-HT_2A_, or 5-HT_2C_ receptor were incubated briefly with respective radiolabeled ligands at concentrations equivalent to the dissociation constant (*K*_*d*_). Radioligands used for the receptors included [3H]8-hydroxy-2-(dipropylamino) tetralin (8-OH-DPAT; 0.90 nM) for the 5-HT_1A_ receptor [3H]ketanserin (0.40 nM) for the 5-HT_2A_ receptor, and [3H]mesulergine (1.4 nM) for the 5-HT_2C_ receptor. Thereafter, the ligand’s displacement by the substance of interest was measured. Specific binding to the target site was defined by subtracting the non-specific binding (measured in the presence of the receptor’s respective competitor in excess) from the total binding measured. The following competitors were used including pindolol (10 μM) for the 5-HT_1A_ receptor, spiperone (10 μM) for the 5-HT_2A_ receptor, and mianserin (10 μM) for the 5-HT_2C_ receptor.

#### 2.6.2 5-HT activation potency

Activation of 5-HT_1A_, 5-HT_2A_, and 5-HT_2B_ receptors by the psilocin metabolites 4-HIAA and 4-HTP was assessed by quantifying the accumulation of inositol monophosphate 1 (IP1) utilizing the Cisbio IP-One G_q_ Kit (Cisbio Bioassays SAS, Codolet, France) following the manufacturer’s instructions. Psilocin and 5-HT were used as comparator and control substances, respectively. NIH/3T3 cells stably expressing the human 5-HT_1A_, 5-HT_2A_, and 5-HT_2B_ receptors were seeded at a density of 3,000 cells (5-HT_1A_ and 5-HT_2A_) or 4,000 cells (5-HT_2B_) in 384-well plates using Opti-MEM medium from Gibco (ThermoFisher, Life Technologies, Zug, Switzerland). Subsequently, the test compounds were introduced, and the plates were incubated for 90 min at 37°C, followed by a 60 min incubation with Anti-IP1-Cryptate and IP1-d_2_ at room temperature. The formation of stimulated IP1 was quantified by homogeneous time-resolved fluorescence (HTRF) measurements using a BioTek Synergy H1 microplate reader (Agilent Technologies, Basel, Switzerland). The obtained raw data for receptor activation were subjected to normalization, wherein the baseline signal was set at 0%, and the maximum signal stimulated by 5-HT at the specific receptor was established as 100%.

### 2.7 Mouse study samples

C57BL/6J adult male mice (Janvier, Le Genest-Saint-Isle, France) were maintained in littermate pairs in standard cages (containing sawdust, a sleeping chamber, tissue paper, and a wood stick) with water and pellet food available *ad libitium*. The colony room was on a reversed light-dark cycle (dark phase 07:00 to 19:00) with temperature at 22°C and humidity at 50–60%. A total of 10 mice were dosed orally with either 3 mg/kg bodyweight psilocybin (n = 5) or saline solution (n = 5). Dosing and blood sampling were conducted between 09:00 and 13:00. The mouse was placed in a plastic restrainer with the tail protruding through a hole in the end of the tube. The tail was immersed in warm water for 1 min for vasodilation and then a small incision was made on the lateral tail surface near the distal tip. 50 μL of blood was massaged gently into an EDTA-coated capillary blood tube (Mircovette, Sarstedt, Nümbrecht, Germany). The first (baseline) blood sample was collected directly before compound administration via oral gavage and the mouse was then returned to the home cage. Further blood samples were collected at 15, 30, 60, and 120 min post-treatment. Each sample was drawn from the same incision site, with mice maintained in the home cage in the intervening periods. The blood samples were stored on ice and centrifuged for 10 min at 780 × g and 4°C. Subsequently, the plasma was transferred into cryotubes (Protein LoBind, Eppendorf) and stored at −80 °C until analysis. All procedures were conducted under a permit for animal experimentation (ZH038/2022) issued by the Veterinary Office Zurich in accordance with the Animal Protection Act (1978) of Switzerland. The use of psilocybin was authorized by the Federal Office of Public Health (FOPH).

Deglucuronidation of psilocin and 4-HIAA in the plasma samples and thus determination of the conjugated metabolite fraction was performed according to a previously described method by Kolaczynska et al. (Kolaczynska et al., 2021) and adapted to the low plasma volumes available.

Pharmacokinetic parameters were calculated using Phoenix WinNonlin software (version 8.1.0, Certara, Princeton, USA). The elimination half-life (t_1/2_, min) was assessed as t_1/2_ = ln(2)/λ, while the elimination constant was calculated by linear regression in the terminal elimination phase. The maximal plasma concentration (C_max_, ng/mL) and the time to reach this concentration (t_max_, h) were direct read-outs of the graphical plots.

### 2.8 Human study samples

A subset (n = 5) of pharmacokinetic study samples from a published double-blind, placebo-controlled, crossover study by Holze et al. (Holze et al., 2022b) was reanalyzed for psilocybin metabolites. The clinical study was approved by the ethics committee of Northwestern and Central Switzerland (EKNZ) and registered at clinicaltrials.gov (ID: NCT03604744). The study was executed according to the Declaration of Helsinki and the International Conference of Harmonization for Good Clinical Practice guidelines. Participants received an oral dose of 30 mg psilocybin and 19 blood samples were drawn in lithium heparin-coated S-Monovette tubes (Sarstedt) over a duration of 24 h post-treatment. Subsequently, blood samples were centrifuged at 1,811 × g for 10 min to yield the plasma. Samples were stored at −80°C before analysis by LC-MS/MS.

### 2.9 Human study samples for genotyping

Human plasma samples for genotyping were obtained from two clinical studies, which were approved by the ethics committee of Northwestern and Central Switzerland (EKNZ) and registered at clinicaltrials.gov (ID: NCT03604744 and NCT04227756). The studies were published by Holze et al. (Holze et al., 2022b) and Ley et al. (Ley et al., 2023), respectively. Demographic data of the studies are displayed in Supplementary Table S4. Genomic DNA was extracted from whole blood using the QIAamp DNA Blood Mini Kit (Qiagen, Hombrechtikon, Switzerland) and an automated QIAcube system. SNP genotyping was performed using commercial TaqMan SNP genotyping assays (LuBio Science, Lucerne, Switzerland). We assayed the following SNPs and respective alleles: CYP2D6*3 (rs35742686, assay: C_32407232_50), CYP2D6*4 (rs3892097, assay: C_27102431_D0, and rs1065852, assay: C_11484460_40), CYP2D6*6 (rs5030655, assay: C_32407243_20), CYP2D6*9 (rs5030656, assay: C_32407229_60), CYP2D6*10 (rs1065852), CYP2D6*17 (rs28371706, assay: C_2222771_A0, and rs16947, assay: C_27102425_10), CYP2D6*29 (rs59421388, assay: C_3486113_20), and CYP2D6*41 (rs28371725, assay: C_34816116_20, and rs16947). CYP2D6 gene deletion (allele *5) and duplication/multiplication (allele *xN) were determined using a TaqMan Copy Number Assay (Hs04502391_cn). Activity scores for CYP2D6 were assigned according to established guidelines (Gaedigk et al., 2008; Crews et al., 2012; Hicks et al., 2013; Hicks et al., 2015; Caudle et al., 2020). The classification into different genotypes was as follows: poor metabolizer (PM, activity score = 0), intermediate metabolizer (IM, activity score = 0.5–1), extensive metabolizer (EM, activity score = 1.5–2), and ultra-rapid metabolizer (UM, activity score > 2).

### 2.10 LC-MS/MS instrumentation and settings

A modular high-performance liquid chromatography (HPLC) system (Shimadzu, Kyoto, Japan) with four pumps (A, B, C, and D) connected to an API 4000 QTRAP or an API 5000 tandem mass spectrometer (AB Sciex, Ontario, Canada) was used to separate and quantify the analytes of interest. Different analytical methods were applied to detect either psilocin and metabolites, CYP metabolites, UGT metabolites, or MAO metabolites. Specific parameters, mass transitions, and retention times of all analytes are summarized in Supplementary Table S1.

The method to analyze psilocin and related metabolites was previously described in detail and adapted from Kolaczynska et al. (Kolaczynska et al., 2021). Analytes were separated on a Symmetry C18 column (3.5 μM, 4.6 × 75 mm, Waters, Milford, USA) using water supplemented with 0.1% formic acid and methanol supplemented with 0.1% formic acid as mobile phases A and B, respectively. The method was expanded to also include 4-HTP, oxidized psilocin (*m/z* 221.0), and norpsilocin (*m/z* 191.0). 4-HTP and 4-HIAA eluted simultaneously but were detected in the positive and negative mode, respectively. Thus, each sample was analyzed by positive and negative ionization.

For the detection of 4-HQ, the same column and mobile phases were used as for psilocin. However, the method’s flow rate and time program were adapted. In brief, the injected sample (2.5 μL) was transported using 10% mobile phase B at a 0.2 mL/min flow rate onto the analytical column. In the first, 0.5 min of each run, the sample was mixed with mobile phase A (0.6 mL/min) within a T-union positioned in front of the analytical column. The total flow rate was then increased to 0.8 mL/min and maintained at this rate until the end of the run (0.5–4.5 min). The concentration of mobile phase B was increased linearly to 95% between 0.5 and 3.0 min and held at this concentration for 1.0 min. For the last 0.5 min, the column was reconditioned with 10% mobile phase B. The HPLC was only connected to the tandem mass spectrometer from 1.0 to 3.0 min and otherwise directly to the waste. 4-HQ and tryptophan-d_5_ (ISTD) were measured by multiple reaction monitoring (MRM) in the positive ionization mode. The mass transitions of tryptophan-d_5_ were summed to improve sensitivity.

The CYP metabolites were quantified by the method of Luethi et al. (Luethi et al., 2019). In brief, the analytes were separated on an Atlantis T3 column (3 μM, 3.0 × 50 mm, Waters). Mobile phase A consisted of water and 0.1% formic acid for positive and negative ionization modes. Mobile phase B in the positive ionization mode consisted of acetonitrile and 0.1% formic acid, while pure acetonitrile was used for analysis in the negative ionization mode.

MAO substrate DMT and its metabolite IAA were analyzed using the bioanalytical method published by Luethi et al. (Luethi et al., 2022). Analyte separation was conducted using a Luna PFP(2) analytical column (3.0 μM, 2 × 50 mm, Phenomenex). Water and methanol, both supplemented with 0.1% formic acid, served as mobile phases A and B, respectively.

The LC-MS/MS system was operated using Analyst software (version 1.7, AB Sciex) and the data were analyzed with MultiQuant software (version 3.0.3, AB Sciex).

## 3 Results

### 3.1 *In vitro* metabolism and receptor interactions

#### 3.1.1 Human liver microsomes

**FIGURE 1:**
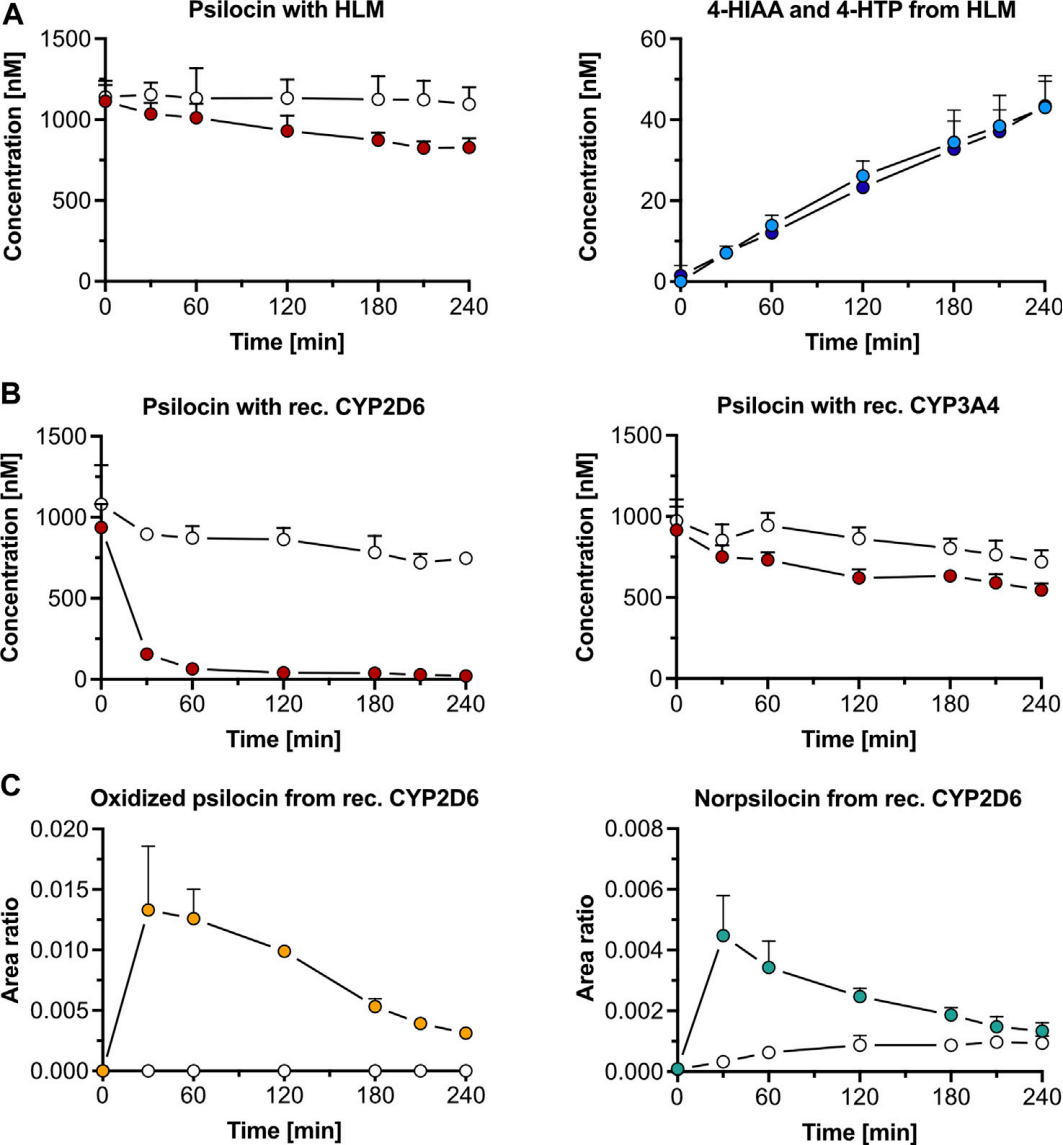
Psilocin’s *in vitro* metabolism with liver microsomes and cytochrome P450 (CYP) enzymes. **(A)** Incubation of 1,000 nM psilocin in the presence (red dots) and absence (white dots) of human liver microsomes (HLM) is depicted on the left. Concurrent 4-hydroxyindole-3-acetic acid (4-HIAA; light blue dots) and 4-hydroxytryptophol (4-HTP; dark blue dots) formation with HLM is shown on the right. **(B)** Incubation of 1,000 nM psilocin with recombinant (rec.) CYP2D6 enzymes (red dots, left) and rec. CYP3A4 enzymes (red dots, right) compared to their respective control conditions (white dots) with selective CYP inhibitors quinidine and ketoconazole, respectively. **(C)** Formation of putative oxidized psilocin (yellow dots, left) and putative norpsilocin (green dots, right) with rec. CYP2D6 enzymes. In the presence of CYP2D6 inhibitor quinidine, the metabolite formation is blocked (white dots).

In the presence of HLM, psilocin concentration (mean ± standard deviation, SD) decreased by 29% from 1,162 ± 146 nM to 829 ± 32 nM after 240 min incubation of 1,000 nM psilocin (Figure 1A). Simultaneously, minor increases in 4-HIAA (43.0 ± 7.9 nM) and 4-HTP (43.4 ± 6.0 nM) concentrations were observed (Figure 1A). In the absence of HLM, no relevant decrease in psilocin concentration (t = 0 min, 1,141 ± 74 nM; t = 240 min, 1,096 ± 105 nM), and no metabolite formation was seen (Figure 1A). Targeted inhibition of specific CYP enzymes present in HLM did not lead to a clear inhibition of psilocin degradation (Supplementary Figure S1). However, CYP-selective substrates, employed as control substances, were metabolized into their corresponding hydroxylated metabolites in the absence of CYP-specific inhibitors. In the presence of CYP-specific inhibitors, these reactions were inhibited (Supplementary Figure S2).

#### 3.1.2 Recombinant cytochrome P450 enzymes

Recombinant CYP2D6 enzymes extensively metabolized 1,000 nM psilocin (mean ± SD) over 240 min (t = 0 min, 937 ± 145 nM; t = 240 min, 21.1 ± 14.2 nM). This reaction was inhibited in the presence of quinidine, a selective CYP2D6 inhibitor (t = 0 min, 1,081 ± 241 nM; t = 240 min, 747 ± 30 nM) (Figure 1B). With recombinant CYP3A4 enzymes, psilocin concentration decreased by 40% (t = 0 min, 916 ± 145 nM; t = 240 min, 546 ± 41 nM). In the presence of the CYP3A4 inhibitor ketoconazole, this reaction was partially blocked as only 26% of psilocin was metabolized (t = 0 min, 973 ± 133 nM; t = 240 min, 720 ± 71 nM) (Figure 1B). Formation of 4-HIAA or 4-HTP was neither observed in the presence of recombinant CYP2D6 nor with CYP3A4 enzymes.

Two putative minor metabolites, an oxidized psilocin metabolite (m/z 221.0 → 176.0 Da) and norpsilocin (*m/z* 191.0 → 160.0 Da), were formed by CYP2D6 (Figure 1C; Supplementary Table S1). The signal intensity (area ratio) of both, oxidized psilocin (t = 30 min, 0.013 ± 0.005 counts; t = 240 min, 0.0030 ± 0.0003 counts) and norpsilocin (t = 30 min, 0.004 ± 0.001 counts; t = 240 min, 0.0010 ± 0.0003 counts), peaked after 30 min and declined again by 240 min. Co-incubation with the CYP2D6 inhibitor quinidine resulted in reduced and delayed formation of norpsilocin, whereas no oxidized psilocin metabolite was detected (Figure 1C).

Furthermore, other recombinant CYPs (1A2, 2B6, 2C8, 2C9, 2C19, and 2E1) did not metabolize psilocin and, consequently, no production of metabolites was observed (Supplementary Figure S3). In the control assays, CYP-specific substrates were metabolized to their respective hydroxylated metabolites. These reactions were inhibited in the presence of CYP-specific inhibitors (Supplementary Figure S4).

#### 3.1.3 Monoamine oxidases

##### 3.1.3.1 Monoamine oxidase A inhibition in human liver microsomes

**FIGURE 2:**
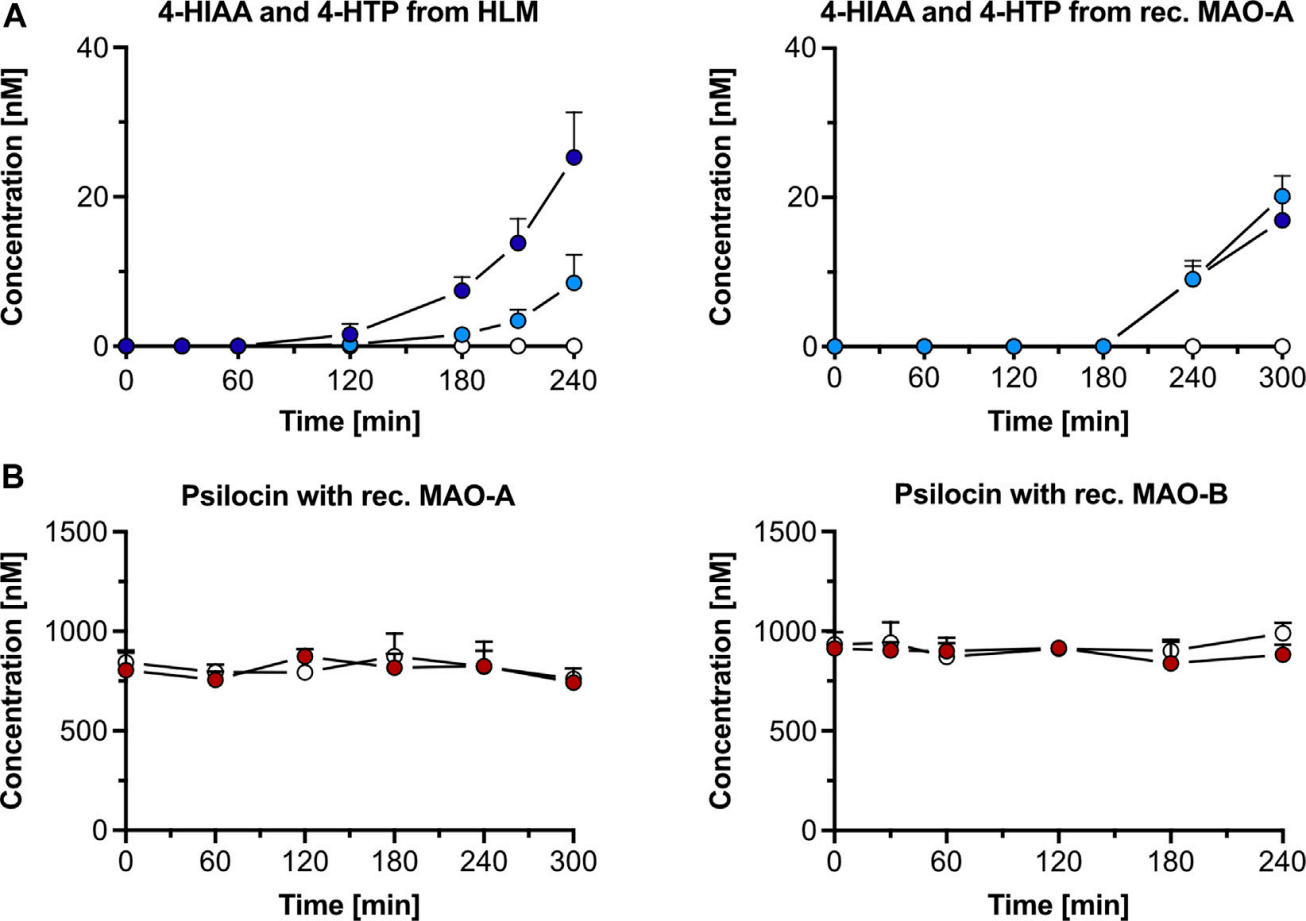
Psilocin’s *in vitro* metabolism with human liver microsomes (HLM) and monoamine oxidase (MAO). **(A)** 4-hydroxyindole-3-acetic acid (4-HIAA; light blue dots) and 4-hydroxytryptophol (4-HTP; dark blue dots) formation after incubation of 1,000 nM psilocin with HLM (left) or recombinant (rec.) MAO-A (right). In the presence of MAO-A inhibitor clorgyline, neither 4-HIAA nor 4-HTP formation was observed (white dots). **(B)** Incubation of 1,000 nM psilocin in the presence of rec. MAO-A (red dots, left) or MAO-B (red dots, right) and in combination with MAO inhibitors (white dots) clorgyline and R-deprenyl, respectively.

Incubation of psilocin (1,000 nM) with HLM and the MAO-A selective inhibitor clorgyline led to complete inhibition of 4-HIAA and 4-HTP formation. Minimal amounts (mean ± SD) of 4-HIAA (t = 240 min, 8.5 ± 3.8 nM) and 4-HTP (t = 240 min, 25.3 ± 6.0 nM) were observed after incubation with HLM alone (Figure 2A). However, in this assay, psilocin concentration did not visibly decrease when incubated with HLM (Supplementary Figure S5A). Furthermore, incubation of 4-HIAA or 4-HTP with HLM did not lead to any biotransformation (Supplementary Figure S5B).

In the control condition, the non-specific MAO substrate kynuramine was readily metabolized to 4-HQ (t = 240 min, 1,974 ± 302 nM) in the presence of HLM. 4-HQ production was inhibited when incubated with the MAO inhibitor clorgyline (t = 240 min, 347 ± 67 nM) (Supplementary Figure S5A).

##### 3.1.3.2 Recombinant monoamine oxidase A and B

Psilocin incubation (1,000 nM) with recombinant MAO-A and MAO-B enzymes did not visibly decrease its concentration (Figure 2B). However, a minor increase in 4-HIAA (20.1 ± 2.8 nM) and 4-HTP (16.9 ± 2.9 nM) concentration was observed after incubation for 300 min with recombinant MAO-A enzymes (Figure 2A). In the presence of the MAO-A inhibitor clorgyline, the formation of both metabolites was inhibited (Figure 2A). Incubation of psilocin with MAO-B did not produce any 4-HIAA or 4-HTP (Supplementary Figure S5C).

In the control conditions, kynuramine was metabolized by MAO-A (t = 0 min, 135,200 ± 23,816 counts; t = 300 min, 48,503 ± 12,251 counts) and MAO-B (t = 0 min, 82,853 ± 11,336 counts; t = 240 min, 9,533 ± 8,311 counts). Formation of the metabolite 4-HQ was observed with MAO-A (t = 300 min, 2,694 ± 264 nM) and MAO-B (t = 240 min, 4,692 ± 891 nM) (Supplementary Figure S5D). The concentration of the MAO substrate DMT decreased when incubated with recombinant MAO-A enzymes (t = 0 min, 907 ± 31 nM; t = 240 min, 110 ± 29 nM), and its metabolite IAA was formed (t = 240 min, 30.3 ± 4.2 nM). The decrease in DMT concentration was almost completely inhibited by clorgyline (t = 0 min, 915 ± 11 nM; t = 240 min, 853 ± 69 nM), and no IAA was detected (Supplementary Figure S6A). DMT only marginally decreased with recombinant MAO-B (t = 0 min, 940 ± 47 nM; t = 240 min, 816 ± 56 nM), and small amounts of IAA were formed (t = 240 min, 11.5 ± 4.5 nM). R-deprenyl inhibited the metabolic decrease of DMT with MAO-B (t = 0 min, 954 ± 73 nM; t = 240 min, 1,008 ± 58 nM), and no IAA was observed (Supplementary Figure S6B).

#### 3.1.4 Glucuronidation

##### 3.1.4.1 Human liver microsomes and human intestinal microsomes

No glucuronidation was observed when psilocin (1,000 nM) was incubated with HLM, while incubation with HIM led to a decrease in concentration (mean ± SD) of 37% after 240 min (t = 0, 1,085 ± 157 nM; t = 240, 684 ± 53 nM) through glucuronidation (Supplementary Figure S7A). In the absence of HIM, only a minor psilocin decrease was observed (t = 0, 980 ± 77 nM; t = 240, 893 ± 91 nM) (Supplementary Figure S7A).

In the control condition, HIM glucuronidated 99% of OH-efavirenz over 240 min (t = 0, 45,300 ± 5,766 counts; t = 240 min, 470 ± 394 counts) (Supplementary Figure S7B).

##### 3.1.4.2 Recombinant UDP-glucuronosyl transferase 1A10

Psilocin concentration (1,000 nM, mean ± SD) remained stable over 240 min in the presence of recombinant UGT1A10 enzymes (t = 0 min, 1,029 ± 60 nM; t = 240 min, 979 ± 95 nM) (Supplementary Figure S7A). Similar findings were observed when psilocin was incubated in the absence of UGT1A10 (t = 0, 980 ± 77 nM; t = 240, 893 ± 91 nM) (Supplementary Figure S7A). However, in the control condition, UGT1A10 glucuronidated 98% of OH-efavirenz over 240 min (t = 0, 41,700 ± 10,936 counts; t = 240 min, 1,007 ± 155 counts) (Supplementary Figure S7B).

#### 3.1.5 5-HT receptor interactions

The interactions of psilocin, 4-HIAA, and 4-HTP with human 5-HT_1A_, 5-HT_2A_, 5-HT_2B_, and 5-HT_2_C receptors are summarized in Supplementary Table S2. Psilocin exhibited high binding affinity at the 5-HT_1A_, 5-HT_2A_, and 5-HT_2C_ receptors (*K*_*i*_ < 136 nM), especially at the 5-HT_2A_ receptor (*K*_*i*_ = 41.1 ± 8.9 nM). In contrast to psilocin, the metabolites 4-HIAA and 4-HTP exhibited no relevant affinity to the examined 5-HT receptors (*K*_*i*_ > 10,000 nM). Binding at the human 5-HT_2B_ receptor was not assessed.

Psilocin showed high activation potency at the 5-HT_1A_ (EC_50_ = 1.7 ± 2.4 nM), 5-HT_2A_ (EC_50_ = 35.4 ± 9.7 nM), and 5-HT_2B_ (EC_50_ = 21.5 ± 178 nM) receptor. 4-HIAA and 4-HTP exhibited no relevant activation at the 5-HT_1A_, 5-HT_2A_, and 5-HT_2B_ receptor (EC_50_ > 10,000 nM). The activation potency at the human 5-HT_2C_ receptor was not assessed.

### 3.2 *In vivo* pharmacokinetics and metabolism

#### 3.2.1 Pharmacokinetics and metabolites in mice

**FIGURE 3:**
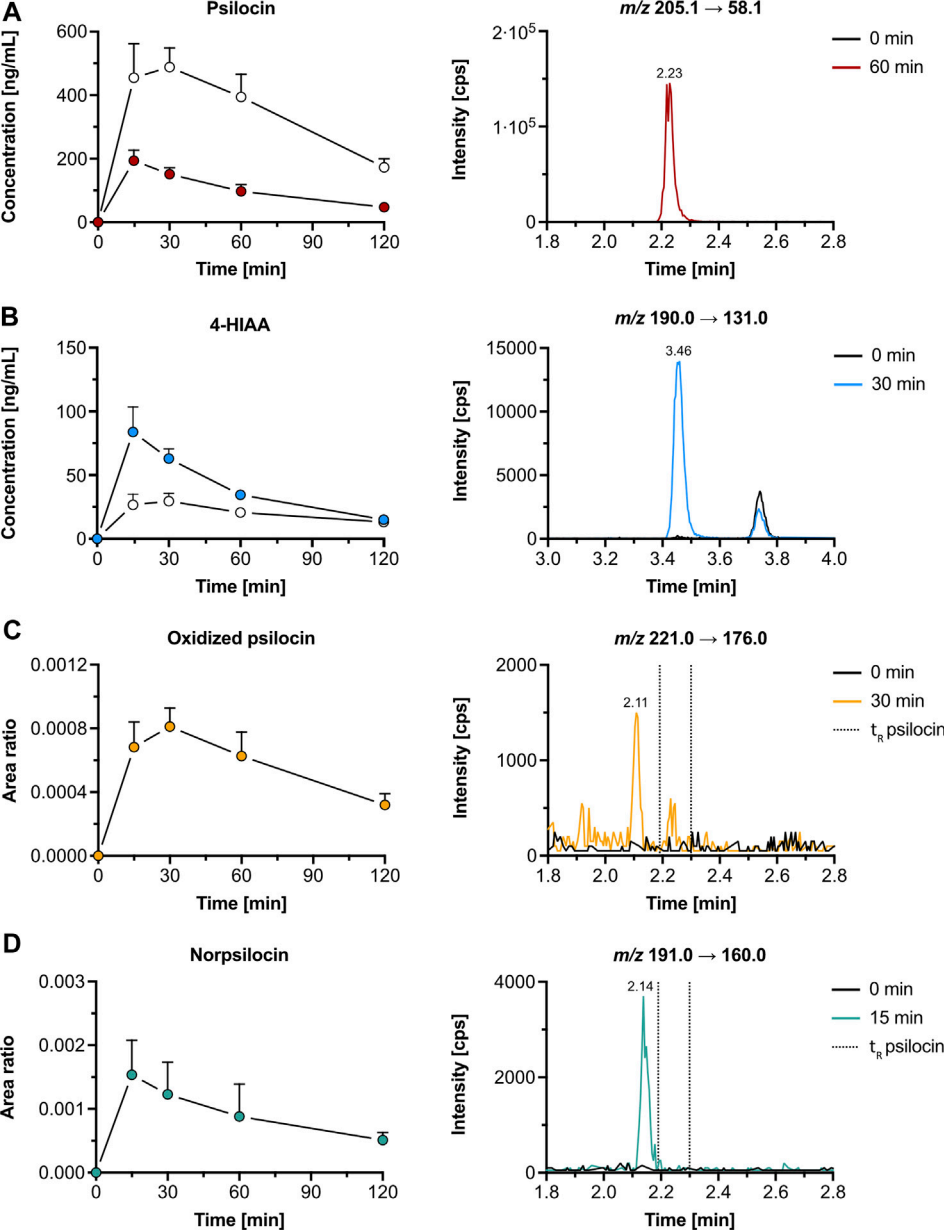
Pharmacokinetics of psilocin and metabolites in mice. Pharmacokinetics (left) of psilocin **(A)**, 4-hydroxyindole-3-acetic acid (4-HIAA; **(B)**), putative oxidized psilocin **(C)**, and putative norpsilocin **(D)** in mice (*n* = 5), following psilocybin administration (3 mg/kg p. o.). On the right, chromatograms recorded in mouse plasma at baseline and close to t_max_ are depicted. Colored dots show the free metabolite concentration (for A and B) or the area ratio (for C and D), while white dots depict the conjugated fraction of the metabolite (for A and B). The area ratio was calculated as the analyte peak area divided by the internal standard peak area (psilocin-d_10_ or tryptophan-d_5_). *m/z*, mass-to-charge ratio; t_R_, retention time.

The average maximal plasma concentration (C_max_, mean ± SD) of psilocin was 198 ± 28 ng/mL after 0.30 ± 0.11 h (t_max_, mean ± SD), while the mean C_max_ of psilocin-O-glucuronide was 2.6-fold higher (521 ± 57 ng/mL) and peaked at 0.35 ± 0.14 h (Figure 3A; Supplementary Table S3). 4-HIAA reached a C_max_ of 84.9 ± 17.7 ng/mL, whilst 4-HIAA-glucuronide displayed a C_max_ of 30.0 ± 6.7 ng/mL. The t_max_ of 4-HIAA and 4-HIAA-glucuronide was 0.30 ± 0.11 h and 0.45 ± 0.11 h, respectively (Figure 3B; Supplementary Table S3). The observed elimination half-life (t_1/2_, mean ± SD) of psilocin and psilocin-O-glucuronide was similar (0.91 ± 0.11 h and 0.97 ± 0.06 h, respectively). 4-HIAA displayed a slightly shorter t_1/2_ of 0.75 ± 0.11 h, while t_1/2_ for 4-HIAA-glucuronide was 1.38 ± 0.27 h (Supplementary Table S3).

In addition to psilocin, 4-HIAA, and their conjugated metabolites, two minor psilocybin metabolites were observed. The oxidized psilocin metabolite had a retention time of 2.11 min and a t_max_ of approximately 0.5 h in mice (Figure 3C). On the other hand, a demethylated psilocin metabolite, likely norpsilocin, with a retention time of 2.14 min was observed (Figure 3D). The peak area of putative norpsilocin increased over time with a t_max_ at approximately 0.25 h post-treatment (Figure 3D). Neither of the aforementioned metabolites was present in blank mouse plasma. Also, psilocybin metabolite 4-HTP could not be observed in the analyzed mouse plasma samples.

#### 3.2.2 Pharmacokinetics and metabolites in human

**FIGURE 4:**
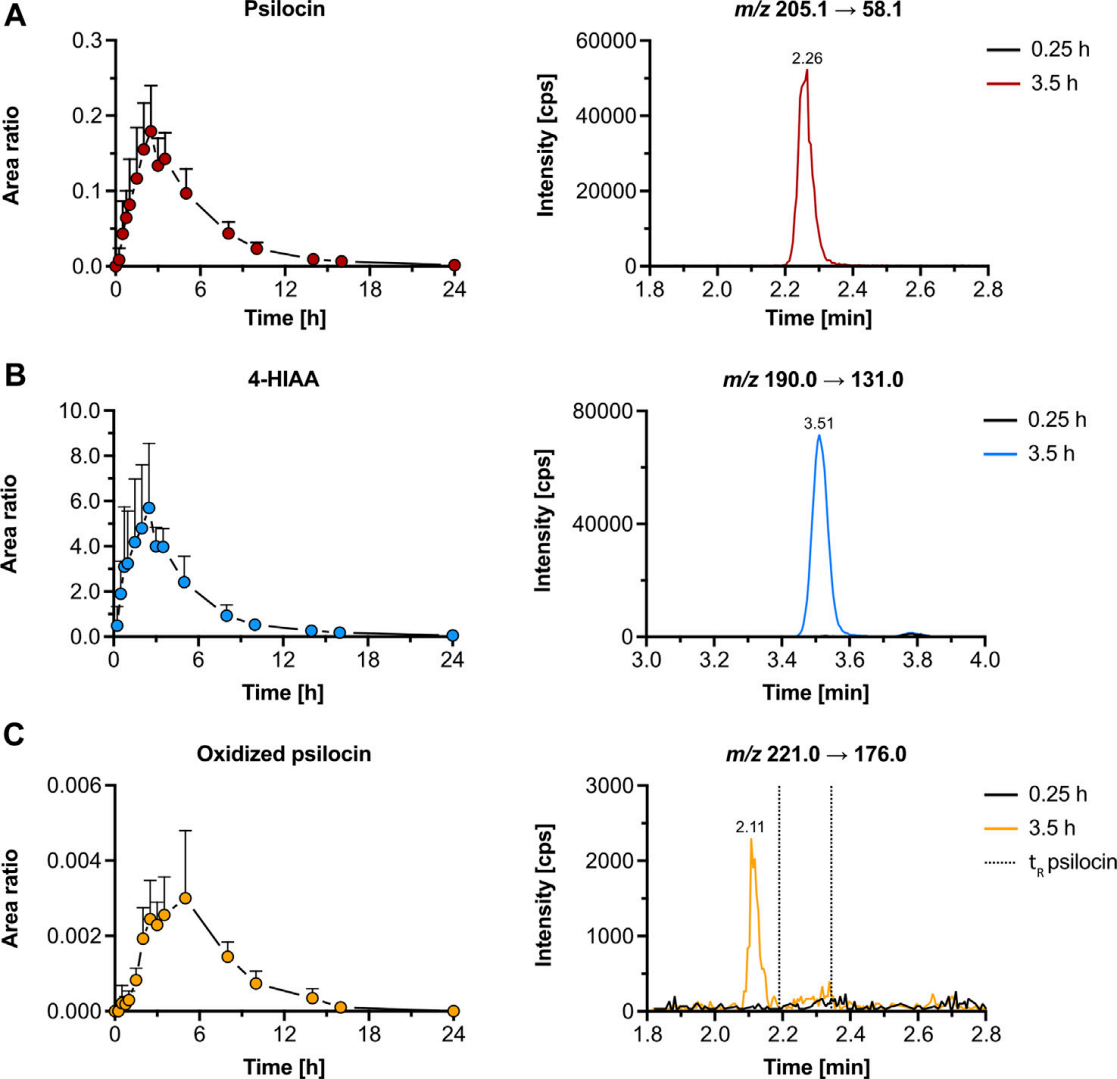
Pharmacokinetics of psilocin and metabolites in humans. Pharmacokinetics (left) of psilocin **(A)**, 4-hydroxyindole-3-acetic acid (4-HIAA; **(B)**), and putative oxidized psilocin **(C)** in humans (*n* = 5), following psilocybin intake (30 mg p. o.). On the right, chromatograms recorded in human plasma at baseline and close to t_max_ are depicted. Colored dots depict the area ratio over time. Detailed pharmacokinetic analysis of psilocin and 4-HIAA in the herein-analyzed human samples was published by Holze et al. (2022a). The area ratio was calculated as the analyte peak area divided by the internal standard peak area (psilocin-d_10_ or tryptophan-d_5_). *m/z*, mass-to-charge ratio; t_R_, retention time.

We reanalyzed psilocybin’s pharmacokinetics in samples of five participants from a study by Holze et al. to assess the presence of the metabolites that we detected *in vitro* (Holze et al., 2022a). In human plasma samples, both psilocin and 4-HIAA reached t_max_ at around 2.5 h post-treatment. Their chromatographic retention was 2.26 min and 3.51 min, respectively (Figures 4A,B). Furthermore, the CYP2D6 genotype did not alter the plasma concentration of free psilocin (Supplementary Figure S8).

Additionally, we were able to detect an oxidized psilocin metabolite with a retention time of 2.11 min (Figure 4C). The metabolite reached t_max_ at 5 h and no signal was present in blank human plasma. Moreover, neither norpsilocin nor 4-HTP was detected in human samples.

## 4 Discussion

In the present study, we elucidated psilocybin’s metabolic pathways *in vitro* by incubating psilocin with a range of human metabolic enzymes, as well as *in vivo* in mice and humans. In addition, we tested the 5-HT receptor binding affinity and activation potency of psilocin and some of its major metabolites. We observed that psilocin is metabolized by HLM and MAO-A to 4-HIAA and 4-HTP. Minor formation of psilocin-O-glucuronide was observed in HIM, but not with recombinant UGT1A10. The putative minor metabolites, norpsilocin, and oxidized psilocin, were formed by human recombinant CYP2D6. In contrast to *in vitro* observations, humans and mice showed significant psilocin glucuronidation and formation of 4-HIAA but not 4-HTP. We confirmed that putative oxidized psilocin was present in both species, while putative norpsilocin was observed solely in mice. Psilocin interacted with 5-HT_1A_, 5-HT_2A_, 5-HT_2B_, and 5-HT_2C_ receptors in the nanomolar range, while the metabolites 4-HIAA and 4-HTP showed no relevant interaction.

**FIGURE 5:**
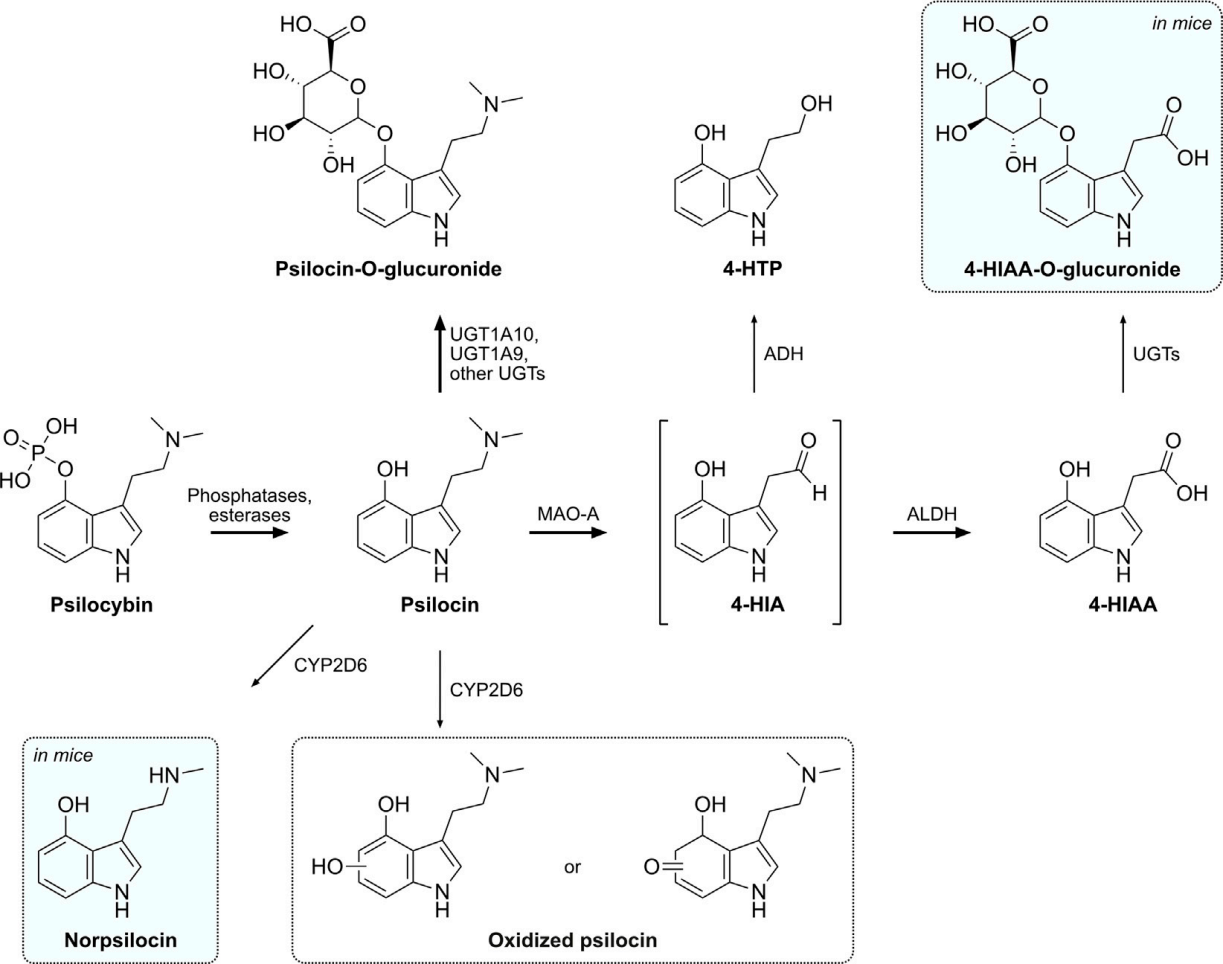
Proposed metabolic pathway of psilocybin and its active metabolite psilocin. Intermediate metabolites are shown in brackets, while putative or not structurally confirmed metabolites are shown in a dotted frame. Metabolites that were only detected in mice are shaded in blue. Major metabolic pathways are marked with bold arrows, while proposed enzymes catalyzing these reactions are indicated next to the arrows. 4-HIA, 4-hydroxyindole-3-acetaldehyde; 4-HIAA, 4-hydroxyindole-3-acetic acid; 4-HTP, 4-hydroxytryptophol; ADH, alcohol dehydrogenase; ALDH, aldehyde dehydrogenase; MAO-A, monoamine oxidase A; UGT, UDP-glucuronosyl transferase.

Based on our findings and the research of others, we propose a metabolic pathway for psilocybin as outlined in Figure 5. Psilocybin is rapidly dephosphorylated to psilocin by alkaline phosphatases and non-specific esterases in the intestines, kidneys, and probably also in the blood circulation (Hasler et al., 1997; Dinis-Oliveira, 2017). In mice, a competitive alkaline phosphatase substrate can occupy most of the enzyme and prevent the conversion of the prodrug psilocybin to psilocin (Horita, 1963). Studies in rats have shown that psilocybin probably undergoes a complete transformation to psilocin before being absorbed and distributed in the blood circulation (Eivindvik et al., 1989). In accordance with these findings, we did not detect psilocybin in the plasma of humans or mice. The plasma elimination half-life of psilocin in humans is approximately 2 h, while mice metabolize psilocin at double this rate, resulting in a half-life of around 0.9 h. Therefore, psilocin is extensively metabolized and can undergo several metabolic reactions.

A major part of psilocin is glucuronidated to psilocin-O-glucuronide and is excreted via urine in the first 24 h. In clinical studies, plasma concentrations of conjugated psilocin were around 4-fold higher than concentrations of free psilocin (Holze et al., 2022a). The half-life of psilocin-O-glucuronide in humans is around 4 h, while in mice it was approximately 4-fold lower (∼1 h) (Holze et al., 2022a). Manevski et al. reported that UGT1A10 has the highest glucuronidation activity for psilocin, while UGT1A9 may also be a main contributor due to its high expression in the liver (Manevski et al., 2010). Contrary to those findings, our *in vitro* glucuronidation systems were not able to recreate the extensive *in vivo* glucuronidation of psilocin. Although the positive control OH-efavirenz was readily glucuronidated, only minor amounts of psilocin were transformed into the conjugated compound by HIM and no visible transformation was observed with recombinant UGT1A10. Therefore, using more complex systems like primary hepatocytes or human spheroid three-dimensional models might be more suitable to investigate *in vivo* phase II metabolism of psilocybin (Ooka et al., 2020).

Psilocin undergoes another major oxidative metabolic pathway similar to the structurally related monoamine neurotransmitter 5-HT (Svensson et al., 1999). MAO enzymes metabolize the tertiary amino group of psilocin to yield the intermediate aldehyde 4-HIA (Hasler et al., 1997). 4-HIA is then rapidly metabolized to either 4-HIAA or 4-HTP (Hasler et al., 1997; Becker et al., 2022). Recombinant MAO-A enzymes as well as HLM were able to produce minor amounts of 4-HIAA and 4-HTP *in vitro,* while selective MAO-A inhibitors reduced this reaction. Neither MAO-B nor any of the assessed CYP enzymes produced 4-HIAA or 4-HTP, suggesting that only MAO-A is involved. However, since MAO-A likely only catalyzes the production of the intermediate 4-HIA, major amounts of 4-HIAA and 4-HTP might only be produced when functional ALDH or ADH enzymes are also present, similar to 5-HT’s metabolism (Svensson et al., 1999; Dinis-Oliveira, 2017). Moreover, careful interpretation of the produced amounts is advised, considering that DMT, a substance well-documented to be rapidly metabolized by MAO *in vivo*, exhibited only slow metabolism *in vitro*, thus resulting in only minor amounts of its metabolite IAA (Luethi et al., 2022; Vogt et al., 2023). Interestingly, although reported before in other studies (Horita, 1963; Hasler et al., 1997), 4-HTP was neither detected in humans nor in mice but only *in vitro* with HLM. Most likely 4-HTP is either rapidly metabolized in humans and mice or not produced at all.

Minor interspecies differences were detected when comparing the results of human with mouse samples. In humans, 4–5-fold higher maximal concentrations of 4-HIAA were found compared to psilocin (Holze et al., 2022a; Becker et al., 2022), while the concentrations in mice were 2-fold lower compared to psilocin. The elimination half-life of 4-HIAA in mice (0.75 h) was more than 2-fold shorter than in humans. Moreover, 4-HIAA is most likely glucuronidated in mice whilst in human samples there was no evidence of glucuronidated 4-HIAA. Although 4-HIAA possesses several glucuronidation sites, we suggest that it undergoes a similar metabolic reaction as 4-hydroxyindole and psilocin in humans to form the O-glucuronide (Manevski et al., 2010). The O-glucuronidation of 4-hydroxyindole in humans is mainly catalyzed by UGT1A6 and UGT1A9 which also could play an important role in this pathway (Manevski et al., 2010). Alternatively, 4-HIAA could undergo acyl-glucuronidation, to form an unstable acyl-glucuronide, or N-glucuronidation (Regan et al., 2010; Kaivosaari et al., 2011).

Out of the eight assessed CYP enzymes, only CYP2D6 and CYP3A4 showed a relevant metabolic activity, suggesting that CYP1A2, 2B6, 2C8, 2C9, 2C19, and 2E1 are unlikely to be involved in psilocybin’s metabolism. Neither 4-HIAA nor 4-HTP formation could be observed with any of the CYP enzymes, confirming that CYPs are not involved in these metabolic pathways. Psilocin can, however, continue to follow another minor metabolic pathway that may be of relevance in the presence of MAO inhibitors (e.g., antidepressants). Several studies suggested a minor oxidative metabolite with an *o*-quinone or iminoquinone structure produced by, e.g., ceruloplasmin, cytochrome oxidases, or non-enzymatically by Fe^3+^ (Blaschko and Levine, 1960; Horita and Weber, 1961b; Horita, 1963; Kovacic, 2009). We hypothesized that CYP enzymes might be involved in this step and investigated this *in vitro*. CYP3A4 showed minor activity in metabolizing psilocin, however, no previously described metabolite could be identified. Estimating the extent of the contribution of CYP3A4 in psilocin’s metabolism is challenging. Although our data implies CYP3A4 involvement, additional investigation is necessary for a more accurate assessment. CYP2D6 was more active than CYP3A4 as it rapidly and completely metabolized psilocin. Subsequently, oxidized psilocin, potentially a quinone-type structure like psilocin iminoquinone (e.g., 4-hydroxy-5-oxo-*N,N-*dimethyltryptamine) or psilocin hydroquinone (e.g., 4,5-hydroxy-*N,N*-dimethyltryptamine) has been detected following incubation of psilocin with CYP2D6. Afterward, the compound has also been detected in human and mouse plasma. This metabolite might have been described as a psilocybin metabolite in mammalian tissue homogenates, but never before in humans (Horita and Weber, 1961b; Horita, 1963; Dinis-Oliveira, 2017). Based on the parent mass of 220.0 Da and the observed fragmentation pattern in the mass spectrometer (*m/z* 221.0 → 176.0 Da), we can postulate that this metabolite possesses an additional oxygen atom on the benzene ring of the indole moiety. However, the exact position of the oxygen cannot be determined solely with tandem mass spectrometry. We can also not exclude that there are several different metabolites present with different hydroxylation or oxidation sites on the indole ring. Similar metabolic findings have been made for structurally related synthetic tryptamines (Bergh et al., 2024). An *o*-quinone structure as proposed by Dinis-Oliveira et al. can be ruled out since this metabolite would have a different molecular weight (218.0 Da) than the metabolite observed in our experiments (Dinis-Oliveira, 2017). Further structural analysis is needed to elucidate the elemental formula and exact position of the oxygen atom.

In addition, another psilocin metabolite was identified in the CYP2D6 assay and subsequently in mouse plasma. The metabolite possesses a molecular mass of 190.0 Da and therefore most likely corresponds to norpsilocin (*N*-methyl-4-hydroxytryptamine). The fragments observed in the mass spectrometer (*m/z* 191.0 → 160.0 Da) suggest that the compound arises through demethylation of the tertiary amine of psilocin. Interestingly, norpsilocin could not be detected in human plasma samples. To the best of our knowledge, this metabolite has not been observed before as a product of psilocybin’s metabolism, but rather as a synthesis or metabolic product of baeocystin, a demethylated analog of psilocybin, present in hallucinogenic mushrooms of the *Psilocybe* genus (Lenz et al., 2017; Sherwood et al., 2020). Norpsilocin was previously suspected to exert psychoactive effects similar to psilocin. It is nearly a full agonist at the mouse and human 5-HT_2A_ receptor in G_q_-dependent calcium flux assays (EC_50_ < 10 nM) but interestingly did not show any psychoactive properties in head-twitch response experiments with mice (Sherwood et al., 2020). This may be due to the inability to cross the blood-brain barrier or rapid degradation of the secondary amine group (Sherwood et al., 2020; Sherwood et al., 2024). Nevertheless, further analysis with NMR or high-resolution mass spectrometry is needed to confirm the structure of this minor psilocin metabolite. Interestingly, the reduction in psilocin concentration observed with CYP2D6 can probably not be solely attributed to an oxidative metabolite or norpsilocin. This suggests the possibility of additional, unidentified metabolites being present.

Furthermore, we showed that genetically determined CYP2D6 activity did not change exposure to psilocin in humans. Thus, CYP2D6 might not be considered to be critically involved in the main metabolism of psilocin in humans and any between-individual differences in CYP2D6 activity are unlikely to influence the response to psilocin in humans. However, the significance of this data may be constrained by the limited number of samples examined. Nevertheless, these preliminary results could have direct clinical implications for psilocybin-assisted therapy and contrast with LSD and MDMA, wherein poor CYP2D6 function significantly increases exposure to these substances and enhances the acute effects (Schmid et al., 2016; Vizeli et al., 2021).

In general, there are only limited data available on the activity of psilocybin’s metabolites at the human 5-HT receptors. Here, psilocin showed sub-micromolar receptor binding affinity at human 5-HT_1A_ (128 ± 33 nM), 5-HT_2A_ (41.1 ± 8.9 nM), and 5-HT_2C_ (136 ± 35 nM) receptors, confirming the results of Rickli et al. (Rickli et al., 2016). Psilocin further showed sub-micromolar activation potencies at the 5-HT_1A_, 5-HT_2A_, and 5-HT_2B_ receptors (<74.5 ± 6.0 nM). However, neither 4-HIAA nor 4-HTP displayed relevant binding affinity at the 5-HT_1A_, 5-HT_2A_, and 5-HT_2C_ receptors (*K*_*i*_ > 10,000 nM) and also no receptor activation potency at the 5-HT_1A_, 5-HT_2A_, and 5-HT_2B_ receptors (EC_*50*_ > 10,000 nM). The inactivity of these metabolites at the human 5-HT receptors indicates that they are not involved in exerting the psychoactive effects in humans, which is in line with the time course of the subjective effects observed in clinical studies (Holze et al., 2022a). The herein-detected oxidative psilocin metabolite has so far not been investigated at human 5-HT receptors due to its uncertain structure.

## 5 Conclusion

In conclusion, this comprehensive study explored the metabolic pathways of psilocin both *in vitro* and *in vivo* and provides new evidence of involved enzymes. In total, we were able to detect six psilocin metabolites. While confirming the glucuronidation of psilocin *in vivo*, we also detected apparent interspecies differences with the glucuronidation of 4-HIAA and the presence of putative norpsilocin in mice compared with humans. While MAO-A was identified as a key enzyme responsible for psilocin’s oxidative transformation to 4-HIAA and 4-HTP, the additional roles of ALDH and ADH still have to be investigated. CYP2D6 and CYP3A4 seem to be involved to a minor extent in psilocin’s metabolism. CYP2D6 produced norpsilocin and a structurally unresolved oxidized metabolite. However, no metabolite was identified with CYP3A4, requiring further investigation into the extent of its role in psilocin’s metabolism. The herein-employed *in vitro* assays assisted in unraveling the metabolism of psilocin but were unable to closely reproduce phase II metabolic reactions of UGT and MAO as observed in humans and mice. Consequently, it is recommended to use and assess more complex hepatocellular assays to further investigate the metabolism of these tryptamines. The major metabolite 4-HIAA and 4-HTP were inactive at human 5-HT receptors but the activity of oxidized psilocin metabolites and norpsilocin remain to be assessed. Inhibition of psilocin inactivation by MAO could potentially augment the metabolic pathway catalyzed by CYP2D6, thereby altering the pharmacodynamics of psilocybin therapy. However, the CYP2D6 genotype did not influence psilocin concentrations in humans. Moreover, glucuronidation of psilocin would likely continue to be the predominant metabolic pathway, rendering MAO inhibition potentially less important.

Finally, our findings on psilocybin’s metabolism contribute to the safety and efficacy of psilocybin therapy by indicating potential drug-drug interactions and helping advance research on psilocybin as a therapeutic agent.

## Data Availability

The raw data supporting the conclusions of this article will be made available by the authors, without undue reservation.
