# Supplementary material for: In vitro and in vivo metabolism of psilocybin’s active metabolite psilocin
Source: Front Pharmacol. 2024 Apr 29;15:1391689. doi: 10.3389/fphar.2024.1391689 (PMC11089204; doi:10.3389/fphar.2024.1391689)
Supplement: Supplementary file 1 [file DataSheet1.pdf]

Supplementary material

to

In vitro and in vivo metabolism of psilocybin's active metabolite psilocin

Jan Thomann[1,2†], Karolina E. Kolaczynska[1,2†], Oliver V. Stoeckmann[1,2], Deborah Rudin[1,2], Patrick Vizeli[1,2], Marius C. Hoener[3], Christopher R. Pryce[4], Franz X. Vollenweider[5], Matthias E. Liechti[1,2*], Urs Duthaler[1,2,6,7]

[1]Division of Clinical Pharmacology and Toxicology, Department of Pharmaceutical Sciences, University of Basel, Basel, Switzerland

[2] Division of Clinical Pharmacology and Toxicology, Department of Biomedicine, University Hospital Basel, Basel, Switzerland

[3] Neuroscience Research, pRED, Roche Innovation Center Basel, F. Hoffmann-La Roche Ltd, Basel, Switzerland

[4] Department of Psychiatry, Psychotherapy and Psychosomatics, Preclinical Laboratory for Translational Research into Affective Disorders, University of Zurich, Zurich, Switzerland

[5] Department of Psychiatry, Psychotherapy and Psychosomatics, Neurophenomenology and Consciousness, University of Zurich, Zurich, Switzerland

[6] Institute of Forensic Medicine, Department of Biomedical Engineering, University of Basel, Basel, Switzerland

[7] Institute of Forensic Medicine, Health Department Basel-Stadt, Basel, Switzerland

[†] These authors have contributed equally to this work and share first authorship

* Correspondence: Matthias E. Liechti, matthias.liechti@usb.ch

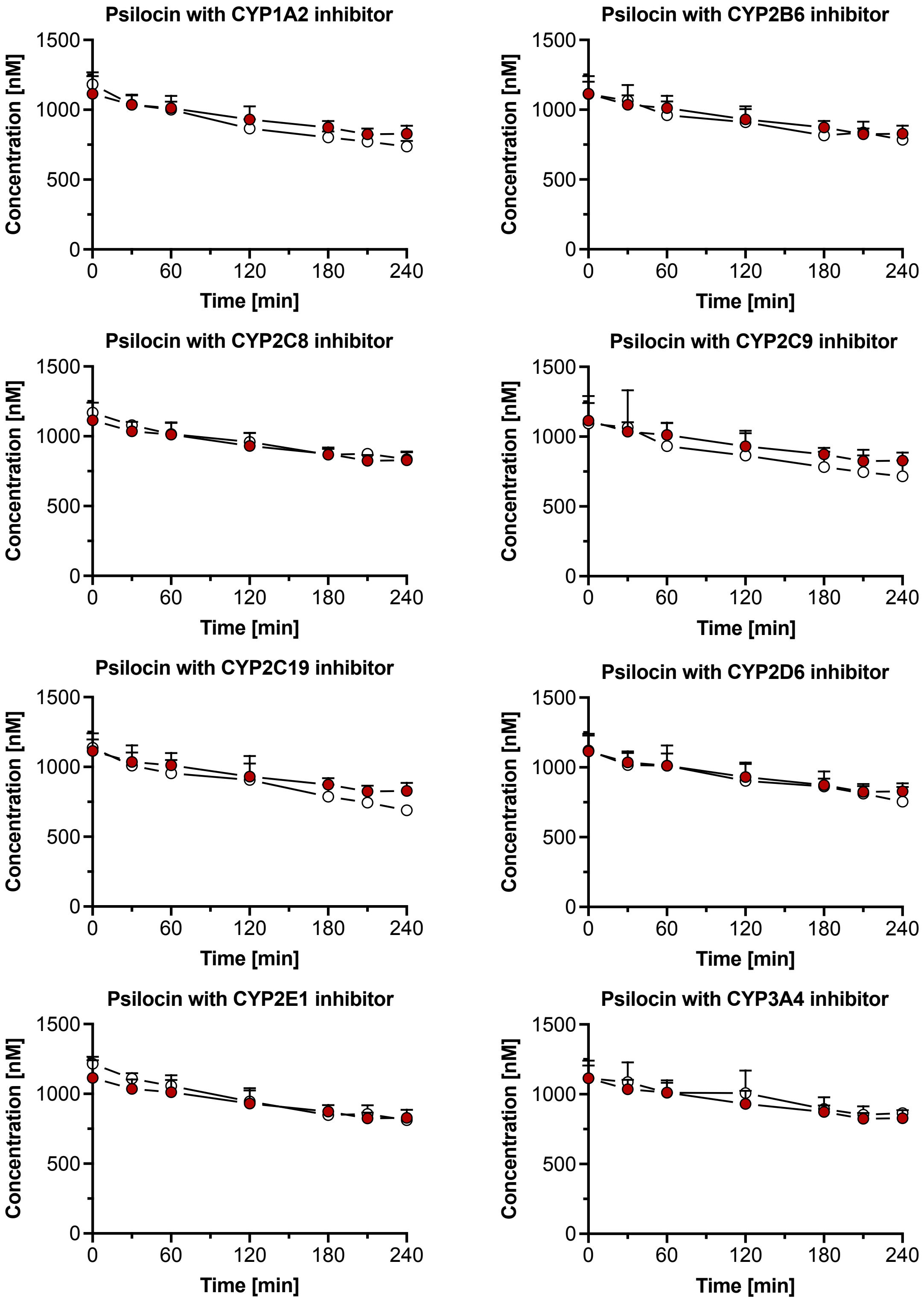

Supplementary Figure S1. Incubation of 1,000 nM psilocin with human liver microsomes (HLM; colored dots) and inhibition of specific cytochrome P450 (CYP) enzymes with selective inhibitors (white dots; CYP1A2, furafylline; CYP2B6, ticlopidine; CYP2C8, montelukast; CYP2C9, sulfaphenazole; CYP2C19, benzylnirvanol; CYP2D6, quinidine; CYP2E1, 4-methylpyrazole; CYP3A4, ketoconazole).

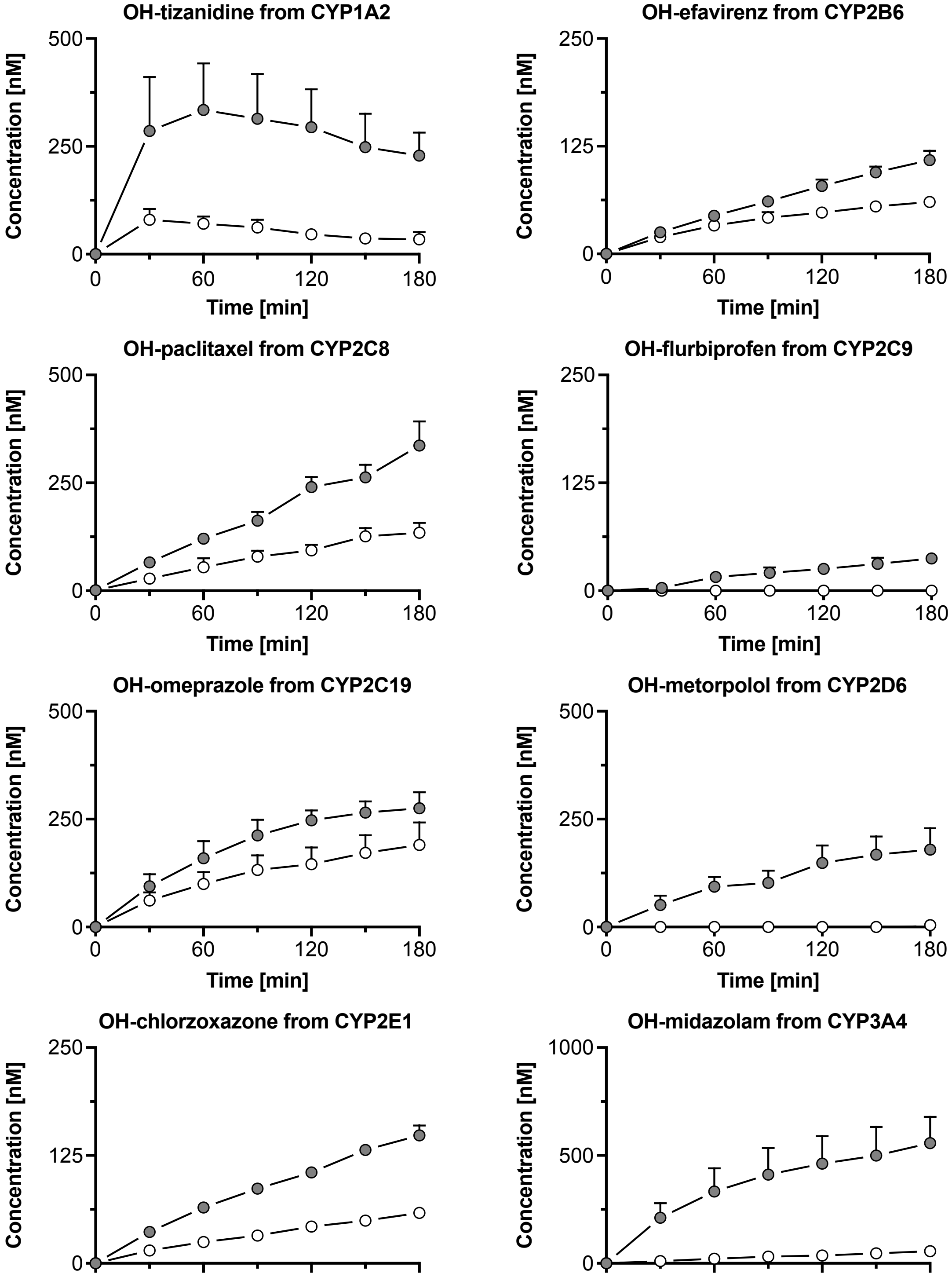

Supplementary Figure S2. Cytochrome P450 (CYP) control assays in human liver microsomes (HLM). CYP substrates (CYP1A2, tizanidine; CYP2B6, efavirenz; CYP2C8, paclitaxel; CYP2C9, flurbiprofen; CYP2C19, omeprazole; CYP2D6, metoprolol; CYP2E1, chlorzoxazone; CYP3A4, midazolam) were incubated with (white dots) and without (grey dots) selective inhibitors (CYP1A2, furafylline; CYP2B6, ticlopidine; CYP2C8, montelukast; CYP2C9, sulfaphenazole; CYP2C19, benzylnirvanol; CYP2D6, quinidine; CYP2E1, 4-methylpyrazole; CYP3A4, ketoconazole). The respective hydroxylated metabolites were quantified to assess assay functionality.

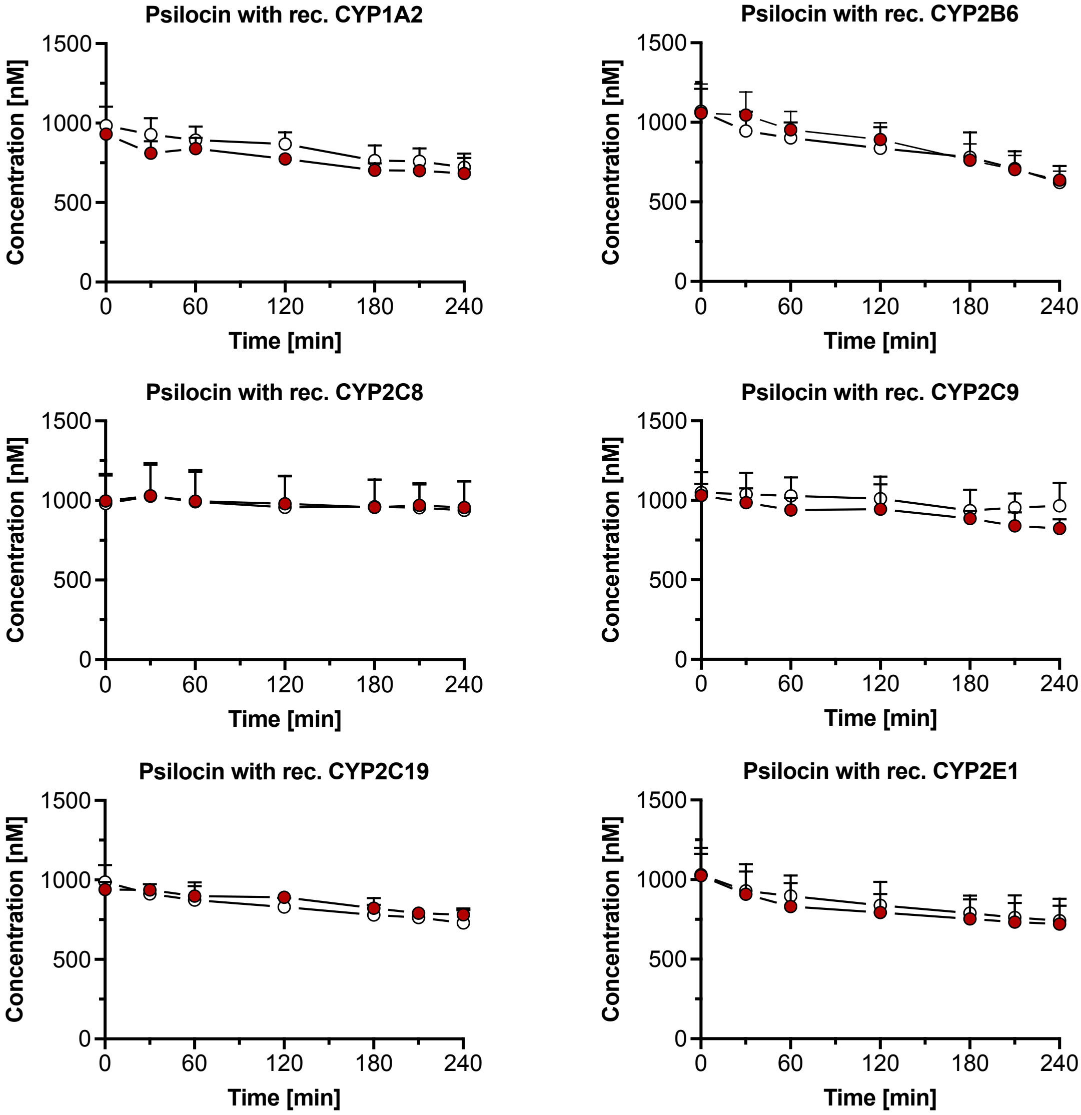

Supplementary Figure S3. Incubation of 1,000 nM psilocin with recombinant (rec.) cytochrome P450 (CYP) enzymes (colored dots) and in combination with a selective inhibitor (white dots; CYP1A2, furafylline; CYP2B6, ticlopidine; CYP2C8, montelukast; CYP2C9, sulfaphenazole; CYP2C19, benzylnirvanol; CYP2E1, 4-methylpyrazole).

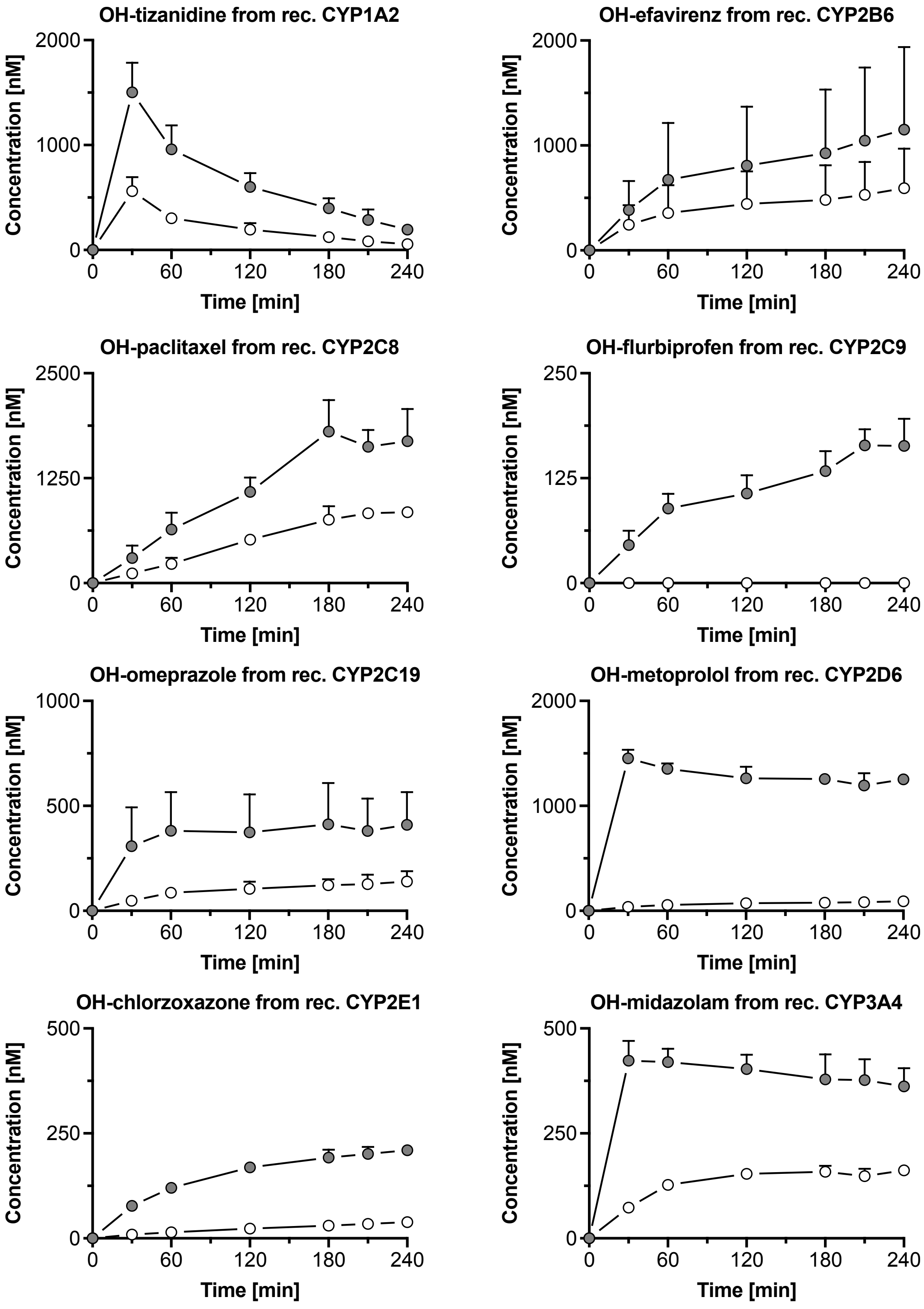

Supplementary Figure S4. Recombinant (rec.) cytochrome P450 (CYP) control assays. CYP substrates (CYP1A2, tizanidine; CYP2B6, efavirenz; CYP2C8, paclitaxel; CYP2C9, flurbiprofen; CYP2C19, omeprazole; CYP2D6, metoprolol; CYP2E1, chlorzoxazone; CYP3A4, midazolam) were incubated with (white dots) and without (grey dots) selective inhibitors (CYP1A2, furafylline; CYP2B6, ticlopidine; CYP2C8, montelukast; CYP2C9, sulfaphenazole; CYP2C19, benzylnirvanol; CYP2D6, quinidine; CYP2E1, 4-methylpyrazole; CYP3A4, ketoconazole). The respective hydroxylated metabolites were quantified to assess assay functionality.

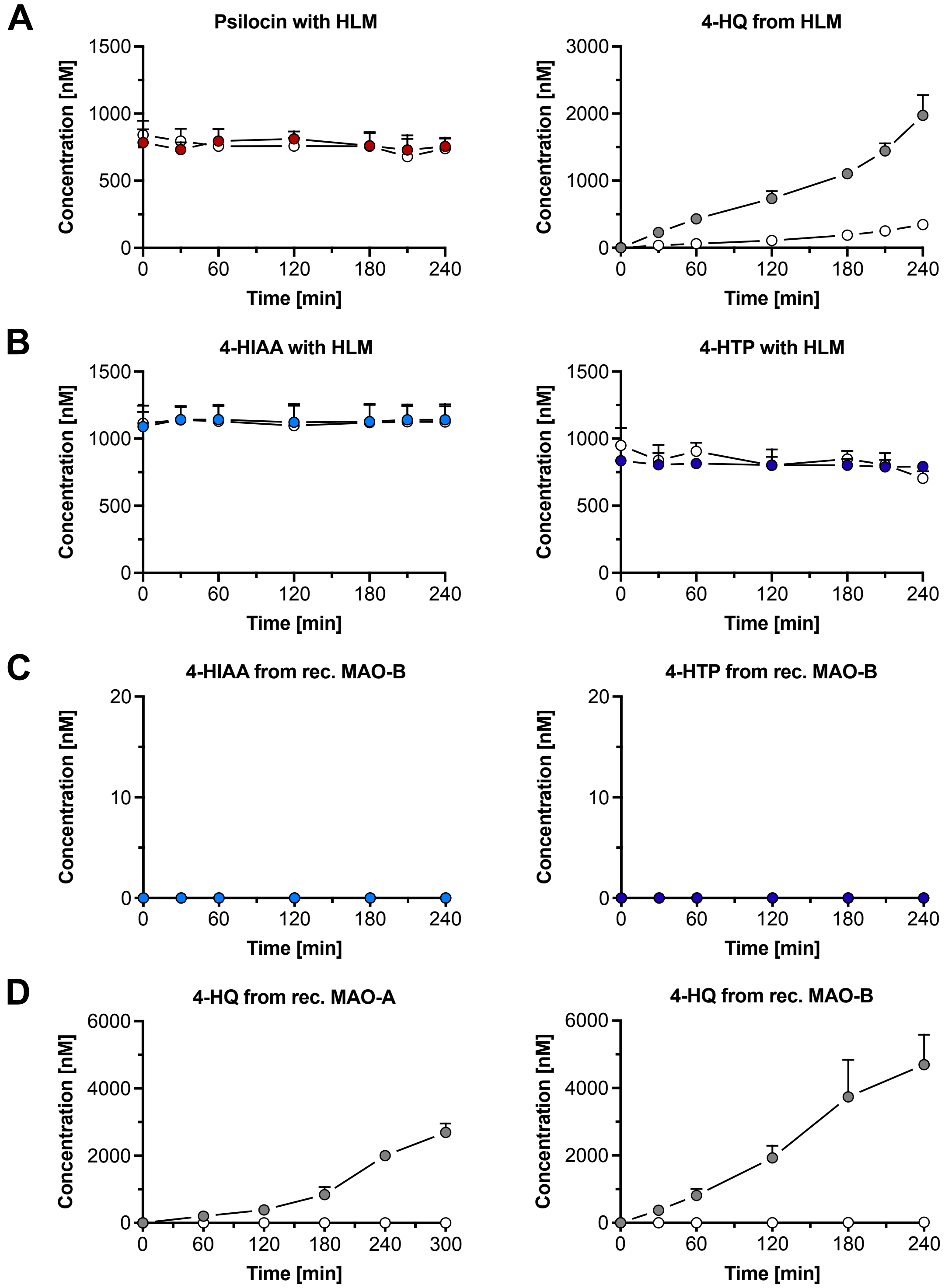

Supplementary Figure S5. A: Incubation of 1,000 nM psilocin with human liver microsomes (HLM; colored dots, left) and 4-HQ formation after incubation of 5 µM kynuramine with HLM (grey dots, right). White dots depict incubation in the presence of monoamine oxidase (MAO) A inhibitor clorgyline. B: Incubation of 1,000 nM 4-hydroxyindole-3-acetic acid (4-HIAA, left) or 1,000 nM 4-hydroxytryptophol (4-HTP, right) with HLM (colored dots) and in the presence of MAO-A inhibitor clorgyline (white dots). C: Neither 4-HIAA (colored dots, left) nor 4-HTP (colored dots, right) formation occurred after incubation of 1,000 nM psilocin with recombinant (rec.) MAO-B enzymes. D: 4-HQ formation after incubation of 5 µM kynuramine with rec. MAO-A (grey dots, left) and rec. MAO-B (grey dots, right). White dots depict incubation in the presence of MAO-A inhibitor clorgyline (left) or MAO-B inhibitor R-deprenyl (right).

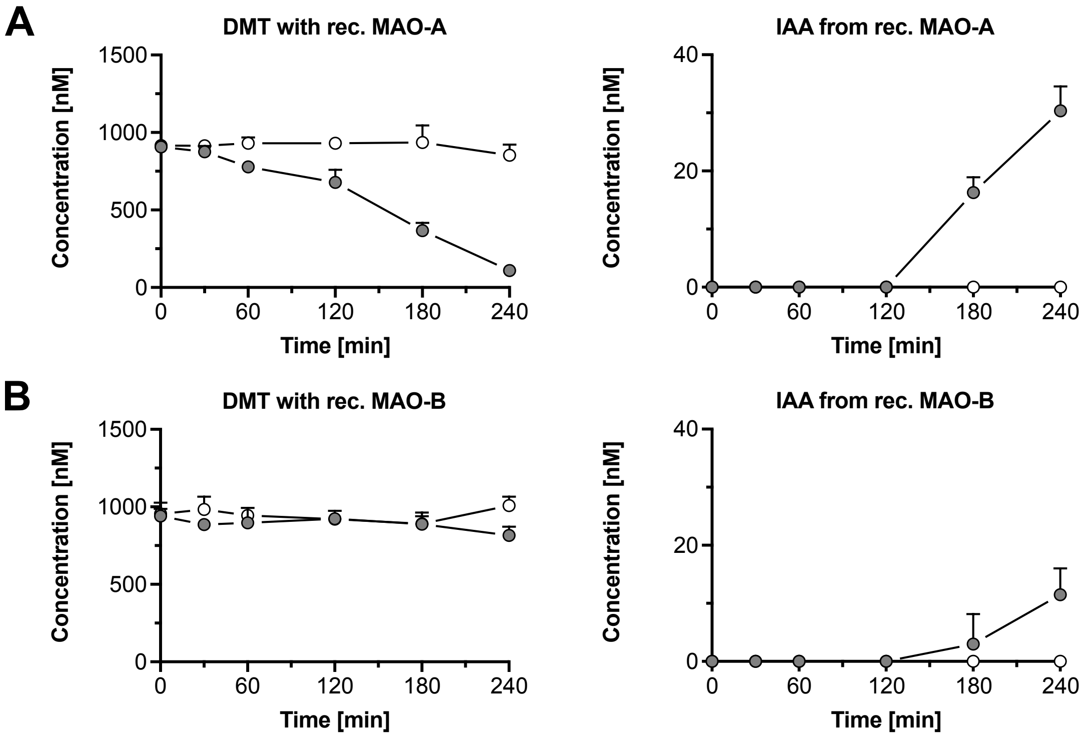

Supplementary Figure S6. A: Incubation of 1,000 nM N,N-dimethyltryptamine (DMT) with recombinant (rec.) monoamine oxidase A (MAO-A) enzymes (grey dots, left) and concurrent formation of metabolite indole-3-acetic acid (IAA; grey dots, right). White dots depict incubation in the presence of MAO-A inhibitor clorgyline. B: Incubation of 1,000 nM DMT with rec. MAO-B enzymes (grey dots, left) and concurrent formation of metabolite IAA (grey dots, right). White dots depict incubation in the presence of MAO-B inhibitor R-deprenyl.

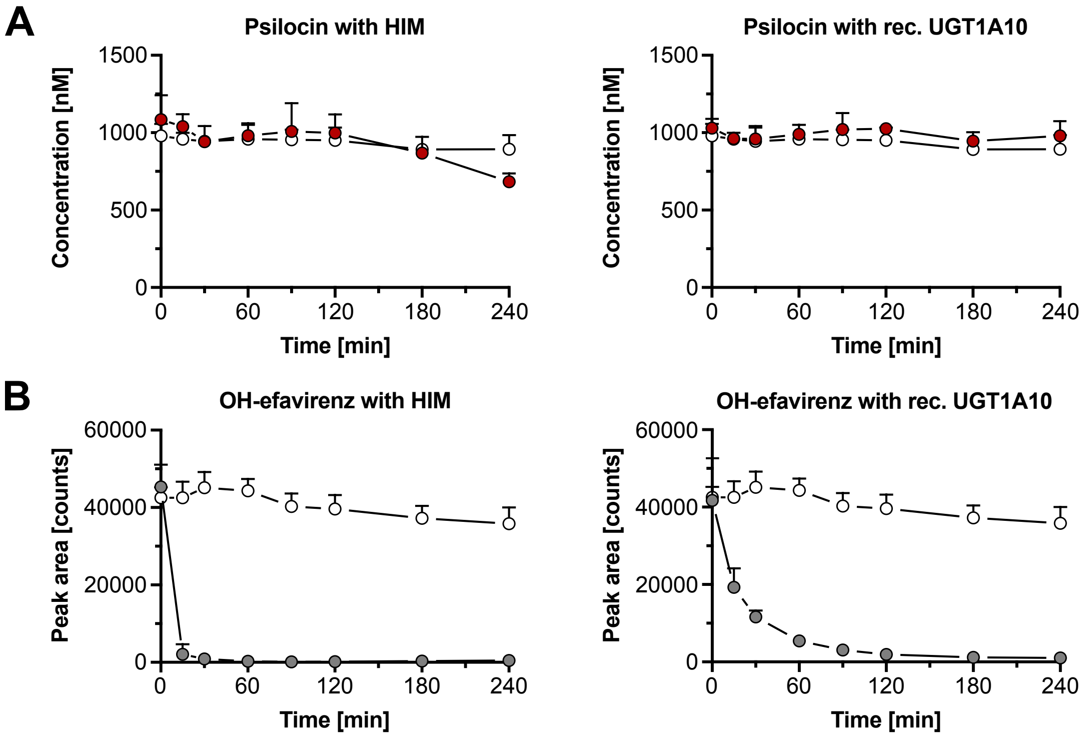

Supplementary Figure S7. Glucuronidation of 1,000 nM psilocin (A) and OH-efavirenz (B) by human intestinal microsomes (HIM; colored dots, left) and recombinant (rec.) UDP-glucuronosyl transferase (UGT) 1A10 (colored dots, right). Incubation of OH-efavirenz in the absence of enzymes is depicted in white dots.

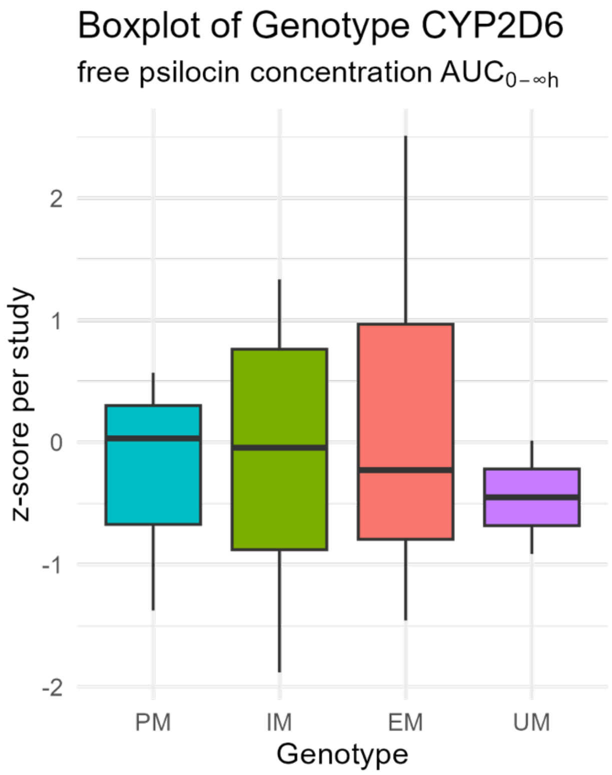

Supplementary Figure S8. The standardized scores (z-transformation) of the area under the time-concentration curve (AUC) of free psilocin in blood plasma from 0 h to infinity of different CYP2D6 genotypes. The z-scores are calculated per study and dose to compare the genotypes across different studies and doses. Poor metabolizer (PM, activity score = 0, n = 3), intermediate metabolizer (IM, activity score = 0.5–1, n = 25), extensive metabolizer (EM, activity score = 1.5–2, n = 58), and ultra-rapid metabolizer (UM, activity score > 2, n = 2).

Supplementary Table S1. Mass spectrometry parameters and calibration ranges of the investigated analytes and their corresponding internal standards.

Analyte	Q1→ Q3 [*m/z*]	Retention time [min]	DP [V]	EP [V]	CE [V]	CXP [V]	Calibration range [nM]
[a]Psilocin	205.2 → 58.1	2.21	36	10	31	10	1–1,000
[a]Psilocin-d_{10}	215.2 → 66.0	2.22	36	10	25	12	NA
[a]4-HTP	178.1 → 160.1	3.48	26	10	17	12	2.5–1,000
[b]4-HIAA	189.9 → 131.0	3.47	-60	-10	-34	-13	2.5–1,000
[a]Oxidized psilocin	221.0 → 176.0	2.11	61	10	27	18	NA
[a]Norpsilocin	191.0 → 160.0	2.14	61	10	27	18	NA
[b]Tryptophan-d_5	208.1 → 119.9	2.87	-105	-10	-24	-7	NA
[c]OH-midazolam	341.9 → 324.0	1.61	106	10	31	8	2.5–1,000
[c]Midazolam-d6	332.2 → 297.2	1.59	106	10	43	28	NA
[c]OH-metoprolol	284.1 → 115.9	1.38	91	10	27	22	10–2,500
[c]Metoprolol-d_6	274.3 → 122.0	1.47	61	10	27	8	NA
[c]OH-omeprazole	361.9 → 214.2	1.50	61	10	17	16	2.5–1,000
[c]Omeprazole-d_3	348.9 → 198.1	1.57	41	10	17	20	NA
[c]OH-paclitaxel I	870.2 → 286.1	2.05	101	10	23	8	5–2,500
[c]OH-paclitaxel II	870.2 → 104.9	2.05	101	10	93	18	5–2,500
[c]Paclitaxel-d_5 I	859.3 → 291.2	2.14	111	10	27	8	NA
[c]Paclitaxel-d_5 II	859.3 → 569.2	2.14	111	10	15	20	NA
[c]OH-tizanidine I	270.0 → 253.1	1.35	91	10	25	18	10–2,500
[c]OH-tizanidine II	270.0 → 60.1	1.35	91	10	35	10	10–2,500
[c]Tizandine-d_4	258.1 → 48.1	1.38	31	10	57	8	NA
[d]OH-efavirenz	330.0 → 357.9	2.10	-75	-10	-30	-13	1–1,000
[d]Efavirenz-d_5	319.0 → 247.8	2.20	-75	-10	-28	-15	NA
[d]OH-flurbiprofen	259.0 → 214.8	1.90	-25	-10	-14	-9	10–2,500
[d]Flurbiprofen-d_3	246.1 → 202.0	2.10	-30	-10	-12	-11	NA
[d]OH-chlorzoxazone	183.7 → 119.8	1.60	-75	-10	-28	-19	10–2,500
[d]Chlorzoxazone-d_3	170.8 → 134.0	1.80	-85	-10	-30	-7	NA
[e]Kynuramine	165.2 → 136.0	1.67	36	10	17	16	NA
[e]4-HQ	146.1 → 77.1	2.58	111	10	41	34	25–5,000
[f]DMT	189.1 → 58.1	1.68	56	10	27	10	0.25–250
[f]DMT-d_6	195.0 → 64.1	1.68	31	10	29	10	NA
[f]IAA	176.0 → 130.1	3.31	56	10	61	8	25–25,000
[f]IAA-d_2	178.0 → 132.1	3.31	56	10	47	8	NA

The sensitivity of OH-paclitaxel, paclitaxel-d_5, OH-tizanidine, kynuramine, and 4-hydroxyquinoline (4-HQ) was improved by the summation of two MS/MS transitions which are indicated as I or II. 4-HIAA, 4-hydroxyindole-3-acetic acid; 4-HTP, 4-hydroxytryptophol; CE, collision energy; CXP, collision cell exit potential; DMT, *N,N*-dimethyltryptamine; DP, declustering potential; EP, entrance potential; IAA, indole-3-acetic acid; MRM, multiple reaction monitoring; *m/z*, mass-to-charge ratio; NA, not assessed; V, voltage. Table adapted from Luethi et al. [1].
[a] measured with MRM using positive ionization method for psilocybin metabolites, [b] measured with MRM using negative ionization method for psilocybin metabolites, [c] measured with sMRM using positive ionization method for CYP metabolites, [d] measured with sMRM using negative ionization method for CYP metabolites, [e] measured with MRM using positive ionization method for MAO metabolites, and [f] measured with MRM using positive ionization method for DMT metabolites.

Supplementary Table S2. Interaction of psilocybin's metabolites with human serotonin receptors.

Analyte	h5-HT_{1A}		h5-HT_{2A}		h5-HT_{2B}		h5-HT_{2C}	
	**Receptor binding**	**Activation potency**	**Receptor binding**	**Activation potency**	**Receptor binding**	**Activation potency**	**Receptor binding**	**Activation potency**
	K_i ± SD [nM]	EC_{50} ± SD [nM]	K_i ± SD [nM]	EC_{50} ± SD [nM]	K_i ± SD [nM]	EC_{50} ± SD [nM]	K_i ± SD [nM]	EC_{50} ± SD [nM]
Psilocin	128 ± 33	1.7 ± 2.4	41.1 ± 8.9	35.4 ± 9.7	NA	21.5 ± 178	136 ± 35	NA
4-HIAA	> 10,000	> 10,000	> 10,000	> 10,000	NA	> 10,000	> 10,000	NA
4-HTP	> 10,000	> 10,000	> 10,000	> 10,000	NA	> 10,000	> 10,000	NA

Values are shown as mean ± standard deviation (SD). 4-HIAA, 4-hydroxyindole-3-acetic acid; 4-HTP, 4-hydroxytryptophol; EC_{50}, half maximal effective concentration; h5-HT, human serotonin receptor, K_i, inhibitory constant; NA, not assessed.

Supplementary Table S3. Pharmacokinetic parameters of psilocybin's metabolites in mouse plasma after administration of 3 mg/kg psilocybin p.o.

**Analyte**	**$t_{1/2}$** [hours]	**t_{max}** [hours]	**C_{max}** [ng/mL]
Psilocin	0.91 ± 0.11	0.30 ± 0.11	198 ± 28
Psilocin-O-glucuronide	0.97 ± 0.06	0.35 ± 0.14	521 ± 57
4-HIAA	0.75 ± 0.11	0.30 ± 0.11	84.9 ± 17.7
4-HIAA-glucuronide	1.38 ± 0.27	0.45 ± 0.11	30.0 ± 6.7

Values are shown as mean ± standard deviation (SD). 4-HIAA, 4-hydroxyindole-3-acetic acid; C_{max}, maximal concentration; $t_{1/2}$, elimination half-life; t_{max}, time to reach maximal concentration.

Supplementary Table S4. Demographics of the study population in the clinical studies used for CYP2D6 genotyping.

	Study NCT03604744	Study NCT04227756
Psilocybin dose, mg	15, 30	20
Subjects, n	28	32
Female, n [%]	14 [50]	16 [50]
Age, years [range]	34 ± 9 [25–52]	29 ± 4 [25–44]
Weight, kg [range]	72 ± 12 [55–104]	71 ± 10 [52–90]
CYP2D6 genotype, (PM, IM, EM, UM)	0, 9, 18, 1	3, 7, 22, 0

Values for age and weight are shown as mean ± standard deviation (SD). Poor metabolizer (PM, activity score = 0), intermediate metabolizer (IM, activity score = 0.5–1), extensive metabolizer (EM, activity score = 1.5–2), and ultra-rapid metabolizer (UM, activity score > 2).

References

[1] D. Luethi, M.C. Hoener, S. Krähenbühl, M.E. Liechti, U. Duthaler, Cytochrome P450 enzymes contribute to the metabolism of LSD to nor-LSD and 2-oxo-3-hydroxy-LSD: Implications for clinical LSD use, Biochem Pharmacol 164 (2019) 129-138.
